# *Mycobacterium tuberculosis* curli pili (MTP) and heparin-binding hemagglutinin adhesin (HBHA) facilitate regulation of central carbon metabolism, enhancement of ATP synthesis and cell wall biosynthesis

**DOI:** 10.1007/s00203-025-04352-w

**Published:** 2025-05-28

**Authors:** T. J. Naidoo, S. Senzani, R. Singh, B. Pillay, M. Pillay

**Affiliations:** 1 Medical Microbiology, School of Laboratory Medicine and Medical Sciences, College of Health Sciences, University of KwaZulu- Natal, Doris Duke Medical Research Institute, 1st Floor, Congella, Private Bag 7, Durban, 4013 South Africa; 2 Department of Medical Microbiology, National Health Laboratory Service, College of Health Sciences, University of KwaZulu- Natal, 1st Floor, Congella, Private Bag 7, George Campbell BuildingDurban, 4013 South Africa; 3 Microbiology, School of Life Sciences, College of Agriculture, Engineering and Science, University of KwaZulu- Natal, Westville Campus, Private Bag X54001, Durban, 4000 South Africa

**Keywords:** *M. tuberculosis* curli pili, Heparin-binding hemagglutinin adhesin, Transcriptomics, ATP synthase, *M. tuberculosis* adhesins

## Abstract

Functional ‘omics’ studies previously identified the *M. tuberculosis* surface located adhesins, heparin-binding hemagglutinin adhesin (HBHA) and curli pili (MTP) as significant potential targets for the design of tuberculosis (TB) point-of-care diagnostics, effective drugs, and vaccines. Little is known on the effect of these adhesins on the pathogen’s transcriptome. The current study, via transcriptomics, elucidated whether the deletion of the single genes, *hbhA* and *mtp*, and double genes, *hbhA*-*mtp*, via specialised transduction, affected global bacterial gene expression. RNA sequencing of *M. tuberculosis* wild-type V9124 (WT), single and double deletion HBHA and MTP mutant strains were confirmed by reverse transcription quantitative polymerase chain reaction (RT-qPCR) on selected genes, and a functional bacterial ATP bioluminescence assay*.* The 43 significantly differentially expressed genes amongst the deletion mutants were functionally categorized into central carbon metabolism (CCM), cell wall biosynthesis and cell wall transport and processes. The increased expression of genes associated with ATP synthase and cell wall processes were confirmed by RT-qPCR. In the absence of the adhesins, a decreased ATP concentration was observed suggesting either increased utilization or alterations to the proton motive force (PMF) that resulted in a potential inhibition of ATP synthesis. Therefore, deletions of the *mtp* and *hbhA* genes were associated with significant perturbations in CCM regulation/function, and transport of proteins to the cell wall, indicating the significant contribution of these adhesins in fundamental processes contributing to TB pathogenesis. Thus, this study indicates that MTP and HBHA influence gene expression in *M. tuberculosis* and represent important targets for TB diagnostic/therapeutic interventions and should be investigated as vaccine and adjunctive therapies.

**Supplementary Information:**

The online version contains supplementary material available at 10.1007/s00203-025-04352-w.

## Introduction

Tuberculosis (TB), caused by *Mycobacterium tuberculosis* (*M. tuberculosis*), remains one of the most devastating bacterial causes of human morbidity and mortality, particularly in low/middle-income countries (Cahill et al. 2020; WHO 2024) with high incidence rates ranging from 150 to 400 per 100,000 population (WHO 2024). These high burdens have been fuelled by a lack of accurate, rapid point of care TB diagnostics, effective drugs for the rapidly evolving drug resistant strains and efficacious vaccine for prevention of adult and adolescent TB (WHO 2024). The slow replication rate of *M. tuberculosis* (WHO 2024) leads to diagnostic delays that significantly impede timely treatment initiation, thus contributing to increased TB transmission (El-Sony et al. 2002), including multi- and extensive drug resistant TB (MDR-TB/XDR-TB) (WHO 2020). The development of improved, rapid TB diagnostics, vaccines, and therapeutics has been hindered largely due to the dearth of accurate, new biomarkers. *M. tuberculosis* harbours multiple adhesins, including the 28-kDa heparin-binding hemagglutinin adhesin (HBHA) (Pethe et al. 2001) and 4-kDa *M. tuberculosis* curli pili (MTP).

HBHA, encoded by *hbhA* (*Rv0475*), is present during the early stages of infection and is responsible for the initial interaction with epithelial cells (Menozzi et al. 2006; Esposito et al. 2011) and THP-1 macrophages (Menozzi et al. 2006), and subsequently, the dissemination of *M. tuberculosis* from the site of infection in mice (Pethe et al. 2001). MTP is encoded by *mtp* (*Rv3312A*) (Alteri et al. 2007), a conserved gene that is present only in *M. tuberculosis* complex strains (Naidoo et al. 2014). The sera of active TB patients were reported to contain anti-MTP IgG antibodies (Alteri et al. 2007; Naidoo et al. 2018). MTP was shown to be involved in biofilm production (Ramsugit et al. 2013) as well as in adhesion and invasion of THP-1 macrophages (Ramsugit and Pillay 2014) and A549 epithelial cells (Ramsugit et al. 2016). MTP was also proposed to modulate cytokine/chemokine induction in epithelial cells as a survival strategy (Ramsugit et al. 2016).

The omics approach has been successfully applied to identify molecules that contribute to key functions during the host–pathogen interactions (Otchere et al. 2024), and which can be investigated as novel biomarkers for effective TB intervention strategies. Transcriptomics (Dlamini 2016; Nyawo 2016) and metabolomics (Ashokcoomar et al. 2020, 2021; Reedoy et al. 2020) elucidated the role of the MTP adhesin in TB pathogenesis. Similarly, functional genomics (Menozzi et al. 2006), proteomics (Shin et al. 2006; Esposito et al. 2011), transcriptomics (Kuvar 2016) and immunological studies (Chiacchio et al. 2017) provided evidence that HBHA is a significant contributor to TB pathogenesis. Functional genomics demonstrated the combined impact of HBHA and MTP in facilitating bacterial growth and biomass formation (Govender et al. 2018, unpublished). Moreover, a transcriptomic study highlighted that MTP and HBHA, in combination, induced transcriptional changes to favour adhesion and subsequent invasion of macrophages (Moodley 2018, unpublished). Collectively, these studies indicated the significant role HBHA and MTP play in *M. tuberculosis* and the suitability of these adhesins as targets for novel vaccine and drug development. However, their specific role in modulating transcriptional changes and global expression of genes with respect regulation of *M. tuberculosis* metabolism, to favour growth and replication of *M. tuberculosis* remains unknown*.* Therefore, the present study used functional transcriptomics, supported by reverse transcription quantitative PCR (RT-qPCR) and a bioluminescence assay, to determine the individual and combined effects of MTP and HBHA on the bacterial transcriptome of *M. tuberculosis* and their regulatory role in metabolic pathways, as well as identify novel pathogen biomarkers.

## Materials and methods

### Ethics approval

The study was approved by the Biomedical Research Ethics Committee (BE383/18).

### Bacterial isolates and growth conditions

The bacterial strains included *M. tuberculosis* wild-type (WT) V9124, a clinical isolate of the F15/LAM4/KZN family previously isolated in Medical Microbiology, University of KwaZulu-Natal, from Tugela Ferry (KwaZulu-Natal, South Africa) (Gandhi et al. 2006), and *mtp* and *hbhA* single and double gene knockout mutants and the corresponding complemented strains constructed in the WT (Table 1).

**Table 1:** Bacterial strains used in this study

Strains	Genetic information	References
WT	Wild-type V9124 (F15/LAM4/KZN), expressing MTP	(Gandhi et al. 2006)
Δ*mtp*	*mtp* deletion mutant, MTP adhesin deficient	(Ramsugit et al. 2013)
*mtp-*complement	*mtp* complemented strain, MTP overexpressing	(Ramsugit et al. 2013)
Δ*hbhA*	*hbhA* deletion mutant, HBHA adhesin deficient	(Govender et al. 2018, unpublished)
*hbhA-*complement	*hbhA* complemented strain, HBHA overexpressing	(Govender et al. 2018, unpublished)
Δ*mtp-hbhA*	*mtp-hbhA* double deletion mutant, MTP and HBHA deficient	(Govender et al. 2018. unpublished)
*mtp-hbhA-*complement	*mtp-hbhA* complemented strain, MTP-HBHA overexpressing	(Sanisha Muniram, Medical Microbiology, UKZN)

*WT* wild-type, *∆mtp mtp*-gene knockout mutant, *∆hbhA hbhA*-gene knockout mutant, *∆mtp-hbhA mtp-hbhA* gene knockout mutant

Briefly, the Δ*mtp* (Ramsugit et al. 2013) and Δ*hbhA* (Govender et al. 2018, unpublished) strains were constructed from *M. tuberculosis* V9124 via specialized transduction, whereby an allelic exchange substrate (AES) replaced specific genes with a hygromycin-resistance (HygR)-sacB cassette (Bardarov et al. 2002). The *mtp*-complemented (Ramsugit et al. 2013) and *hbhA-*complemented (Govender et al. 2018, unpublished) strains were constructed via electrotransformation by insertion of the non-integrating pMV261 plasmids (Bardarov et al. 2002) containing either *mtp* or *hbhA* genes, respectively. The Δ*mtp-hbhA* (Govender et al. 2018, unpublished) was constructed by specialized transduction using the unmarked Δ*hbhA* single deletion mutant and *mtp* high-titre phage containing the targeted gene-specific AES (Bardarov et al. 2002). The *mtp-hbhA* complemented strain (Sanisha Muniram, Medical Microbiology, UKZN) was constructed via electrotransformation using electrocompetent cells and the pMV261-*mtp-hbhA* plasmid (Supplementary Information (SI) Figure S1) (Bardarov et al. 2002). The strains were confirmed by polymerase chain reaction (PCR) using genomic deoxyribonucleic acid (DNA) extracted via InstaGene Matrix (Bio-Rad Laboratories, Hercules, California, USA).

Three technical replicates of each of the seven *M. tuberculosis* strains (*M. tuberculosis* WT V9124, *Δmtp*, *mtp*-complement, *ΔhbhA*, *hbhA*-complement, *Δmtp-hbhA*, and *mtp-hbhA*-complement) were cultured for RNA extraction in three separate biological assays. All strains were cultured in 10 mL of Middlebrook 7H9 medium (Difco, Becton–Dickinson, Franklin Lake, New Jersey, USA) supplemented with 10% (v/v) oleic albumin dextrose catalase (OADC) (Difco, Becton–Dickinson, Franklin Lake, New Jersey, USA), 0.5% (v/v) glycerol (Sigma-Aldrich, Missouri, USA), and 0.05% (v/v) tween-80 (Sigma-Aldrich, Missouri, USA). The cultures were incubated in a shaking incubator (I-26 Shaking Incubator, New Brunswick Scientific, Canada) at 1 × g for 7–8 days at 37 °C to an OD_600nm_ of 1.0, equivalent to approximately 1 × 10^8^ colony forming units (CFU)/mL (Larsen et al. 2007). The OD was determined using the Lightwave II Spectrophotometer (Biochrom Ltd, Cambridge, United Kingdom).

### RNA isolation and sequencing

The RNA extraction was performed as per Larsen et al 2007, with the following modifications. Prior to RNA extraction, the samples were treated with 4 M guanidine thiocyanate (GTC) (Thermo Fisher Scientific, Massachusetts, USA) solution equal to the sample volume to prevent any alterations in transcription and to acquire accurate mRNA representation (Stewart et al. 2002; Butcher 2004; Larsen et al. 2007). Additionally, the final wash step, using 75% ethanol (Sigma-Aldrich, Missouri, USA), was performed twice before the resuspension of the RNA pellet in diethyl pyrocarbonate (DEPC) water (Thermo Fisher Scientific, Massachusetts, USA).

The concentrations, purities and integrities of the RNA samples were assessed using the Nanodrop 2000 (Thermo Fisher Scientific, Massachusetts, USA) and 3-(N-morpholino) propane sulfonic acid (MOPS) (Sigma-Aldrich, Missouri, USA) gel, before DNase (Thermo Fisher Scientific, Massachusetts, USA) treatment and sequencing at Omega Bioservices (Norcross, USA). The bacterial RNA was processed using the Illumina 2 × 150 HiSeq × 10 platform to sequence 34–70 million, 125 bp paired-end reads.

### Read alignment and transcript assembly

The quality of the generated reads was assessed using the FastQC toolkit (RRID: SCR_014583) (version 0.11.8; Babraham Bioinformatics, Cambridge, UK), and pre-processed using Trimmomatic (RRID: SCR_011848) (version 0.36). The remaining clean reads were mapped to the custom-built *M. tuberculosis* H37Rv genome index using hierarchical indexing for spliced alignment of transcripts (HISAT version 2.1.0) (RRID: SCR_015530) (overall alignment percentages ranged from 83.5 to 92.5%) to generate 12 binary alignment files (BAM) (Kim et al. 2015; Pertea et al. 2016). The aligned reads were then assembled using the Stringtie (RRID: SCR_016323) (version 1.2.1) assembler against the *M. tuberculosis* H37Rv annotations as a reference. Gffcompare was used to further quantify and annotate the assembled transcripts into known and novel categories (Pertea et al. 2016; Sreenivasamurthy et al. 2017). To determine the expressed transcripts as a gene transfer format (GTF) file, the Stringtie-merge option was used to merge the Stringtie assemblies. The merged output files were annotated in R (version 1.2.1578) using the Ballgown package (Frazee et al. 2015; Das et al. 2018), to obtain fragments per kilobase of transcript per million mapped reads (FPKM) (Frazee et al. 2015; Das et al. 2018).

### Enrichment and statistical analysis

Further analysis was performed on Ballgown at the gene and transcript level to generate *p-*values for the differential expression and the fold changes (FC) between the deletion mutants and the WT. The generated results were filtered using a FC cut-off value ≥ 1.3 and ≤ 0.75 to identify significant genes for pathway analysis through databases such as Kyoto Encyclopaedia of Genes and Genomes (KEGG) (RRID: SCR_012773) (Kanehisa 2019; Kanehisa and Goto 2000). The DEGs were functionally classified using Mycobrowser (version 5) (RRID: SCR_018242) (Kapopoulou et al. 2011) and BioCyc (RRID: SCR_002298) (Caspi et al. 2016; Karp et al. 2019) to assign pathways and functional categories.

### Reverse transcription quantitative polymerase chain reaction (RT-qPCR)

The genotypic validation of the RNA sequencing data using RT-qPCR, was conducted on the seven bacterial strains (Table 1) and used to assess selected gene expression levels as a result of the *hbhA, mtp,* and *mtp-hbhA* gene deletion. Using the RNA sequencing data, 10 genes which were significantly up-regulated were selected, based on differential expression in the ATP synthesis and cell wall biosynthesis and transport pathways, for RT-qPCR analysis, using the primers listed in Table 2. The 16S rRNA was included as the housekeeping gene (Table 2). The FASTA format for each gene was obtained from MycoBrowser (Kapopoulou et al. 2011) and inserted into Primer3Plus (RRID: SCR_003081) (Untergasser et al. 2007) to select the best primer set for each gene. Prior to DNase treatment, as per the manufacturer’s instruction (Thermo Fisher Scientific, Massachusetts, USA), the RNA concentrations for each sample were standardized to 0.1 µg/µL. The High-Capacity cDNA Reverse Transcription Kit (Roche Applied Sciences, Penzberg, Germany) was used to perform the cDNA conversion as per the Manufacturer’s instructions. After the efficiency of each primer set was tested, the RT-qPCR was conducted using the Ssoadvanced Universal SYBR Green Supermix kit (Bio-Rad Laboratories, Hercules, California, USA) in a 10 µL total reaction volume in a QuantStudio Reverse Transcription PCR System (Applied Biosystems, California, USA). The cycling conditions were as follows; holding stage at 95˚C for 3 min, PCR stage of 40 cycles which included, 95 °C for 30 s, 60 °C for 30 s and 75 °C for 30 s. The melt curve analysis was determined at continuous fluorescence set at 90 °C for 1 min, 60 °C for 30 s and 95 °C for 15 s. The gene expression was normalized using the absolute quantification method.

**Table 2:** Gene and primer sequences selected for analysis of gene expression using RT-qPCR

Gene		Primer sequence (5’–3’)
*atpB*	Forward Reverse	gccacctggctcggtatgac gcgcatctgaatggtgatcg
*atpD*	Forward Reverse	atccccgagctgttcaatgc aagacgtggcccttcacacc
*atpF*	Forward Reverse	tgaagtgagcgcgattgtcc gtcacgttcccgcaagacct
*atpE*	Forward Reverse	ctcatcggcggtggactgat tatgccgcctcaaccaaacc
*atpH*	Forward Reverse	tttatctggtgtggcgattt cctgggcctctagttgttct
*lpqV*	Forward Reverse	ctgtgcgcaatggttttg tactcctcctcggtcgattc
*secE2*	Forward Reverse	caaggtgatcgacatcatcg agcttgatgcggtaggtgat
*Rv2477c*	Forward Reverse	atgggggacatcaagatcaa acaacagcagtttgcacagc
*Rv0987*	Forward Reverse	ccgacacgggtacgattttt actgtggtaggcgcaccttt
*Rv0986*	Forward Reverse	accacatcaccttcgatttc gtgggaatcaggttgaaaaa
*16S rRNA*	Forward Reverse	cctacgggaggcagcagt cgtttacggcgtggactac

### Determination of bacterial bioluminescence

Functional confirmation of decreased cellular ATP concentration was performed using a BacTiter-Glo™ Microbial Cell Viability Assay (Promega, Madison, Wisconsin, USA), as per manufacturer’s instructions, using the Glomax®-Multi + Detection System (Promega, Madison, Wisconsin, USA). The ATP concentrations were normalized using OD.

### Statistical analysis

The gene expression data were normalized using 16S rRNA and analysed using the absolute quantification method (ThermoFisher Scientific 2022). Three biological replicates and three technical replicates were performed for the RNA sequencing, gene expression and bioluminescence assay. GraphPad Prism (version 8) (RRID: SCR_000306) (GraphPad Software, La Jolla, California, USA) was used to perform the parametric, unpaired *t-*test analysis to determine the significance values for both the gene expression and bioluminescence assay. All *p-*values ≤ 0.05 were considered statistically significant.

## Results

### Differential expression of genes induced by the deletion of MTP and HBHA

A total of 43 genes were significantly differentially expressed amongst the deletion mutants and functionally categorized into central carbon metabolism, cell wall biosynthesis, and cell wall transport and processes. Relative to the WT, the number of significant up- and down-regulated genes were 13 and six for the ∆*mtp*, eight each for the ∆*hbhA*, and four and six for the ∆*mtp-hbhA* mutant strains, respectively (Tables 3 and 4).

**Table 3:** The statistically significant up-regulated genes induced by the three *M. tuberculosis* deletion mutants relative to the WT

Functional category	∆*mtp*			∆*hbhA*			∆*mtp-hbhA*		
	Gene	FC	*p-*value	Gene	FC	*p-*value	Gene	FC	*p-*value
Intermediatory metabolism and respiration	*ppdK*	1.562	0.028	*fdxA*	3.152	0.044	*moaA1*	1.502	0.010
	*ilvC*	1.318	< 0.001	*csoR*	1.519	0.034	*lpdA*	1.495	0.022
	*icd1*	1.300	0.006	–	–	–	–	–	–
Oxidative phosphorylation	*ctaC*	1.690	0.009	–	–	–	–	–	–
	*atpF*	1.434	0.041	–	–	–	–	–	–
	*atpE*	1.320	0.010	–	–	–	–	–	–
	*Rv1303*	1.375	0.006	–	–	–	–	–	–
Cell wall and cell processes	*lpqV*	1.927	0.014				–	–	–
	*Rv3377c*	1.562	0.028	–	–	–	–	–	–
	*Rv0986*	1.552	0.039	–	–	–	–	–	–
	*secE2*	1.403	0.006	*secE2*	1.708	0.028	–	–	–
	*Rv0988*	1.552	0.039	–	–	–	–	–	–
Pentose phosphate pathway	–	–	–	*pfkB*	2.088	0.005	–	–	–
Regulatory proteins	–	–	–	*devS*	2.224	0.002	–	–	–
	–	–	–	*devR*	2.130	0.010	–	–	–
Virulence. Detoxifications, and adaptation	–	–	–	*TB31.7*	2.248	0.006	–	–	–
Hypothetical proteins	*Rv3857c*	1.327	0.042	*Rv3128c*	1.633	0.046	*ASdes*	1.706	0.015
Proline-glutamate and proline-proline-glutamate (PE/PPE)	–	–	–	–	–	–	*PPE59*	1.694	0.021

*WT* wild-type, ∆*mtp* *mtp*-gene knockout mutant, ∆*hbhA* *hbhA*-gene knockout mutant, ∆*mtp-hbhA* *mtp-hbhA* gene knockout mutant, *FC* fold change (FC ≥ 1.3); *p* ≤ 0.05, – no significant gene reported in respective category

**Table 4:** The statistically significant down-regulated genes induced by the three *M. tuberculosis* deletion mutants relative to the WT

Functional category	∆*mtp*			∆*hbhA*			∆*mtp-hbhA*		
	Gene	FC	*p-*value	Gene	FC	*p-*value	Gene	FC	*p-*value
Intermediatory metabolism and respiration	*serA2*	0.730	0.028	*purA*	0.654	0.030	–	–	–
	*trpD*	0.731	0.005	*Rv0224c*	0.705	0.027	–	–	–
	*moeX*	0.740	0.027	–	–	–	–	–	–
	*mmuM*	0.739	0.022	–	–	–	–	–	–
Cell wall and cell processes	*mtp*	0.041	0.004	*hbhA*	0.062	0.002	*hbhA*	0.042	< 0.001
	–	–	–	*mtp*	0.533	0.001	*mtp*	0.471	0.019
	–	–	–	*cfp2*	0.747	0.018	–	–	–
Insertion sequences and phages	*Rv2814c*	0.558	0.041	–	–	–	*Rv3326*	0.599	0.016
	–	–	–	–	–	–	*Rv2815c*	0.600	0.008
	–	–	–	–	–	–	*Rv3475*	0.634	0.036
Regulatory proteins	–	–	–	*Rv2642c*	0.644	0.038	–	–	–
Virulence. Detoxifications, and adaptation	–	–	–	*vapC5*	0.733	0.034	–	–	–
	–	–	–	*Rv1082*	0.494	0.044	–	–	–
Hypothetical proteins	–	–	–	–	–	–	*Rv2042c*	0.642	0.027

WT wild-type, ∆*mtp* *mtp*-gene knockout mutant, ∆*hbhA* *hbhA*-gene knockout mutant, ∆*mtp-hbhA* *mtp-hbhA* gene knockout mutant, *FC* fold change (FC ≤ 0.75); *p* ≤ 0.05, – no significant gene reported in respective category

### Functional categorisation of significantly up-regulated and down-regulated genes revealed by RNA sequencing

The genes which displayed significant up- and down-regulation between ∆*mtp* and the WT were functionally categorized. The *ppdK, icd1*, and *ilvC* genes were involved in intermediary metabolism and respiration, while *Rv1303, atpE*, *atpF*, and *ctaC* were associated with the oxidative phosphorylation pathway (OPP) and adenosine triphosphate (ATP) synthesis (Table 3)*.* The genes*, Rv0986*, *Rv0988*, *secE2*, *Rv3377c* and *lpqV* were categorized into cell wall processes, and *Rv3857c* into hypothetical proteins (Table 3). Significant down-regulation was observed in genes involved in several functional groups including *mmuM*, *moeX*, *trpD*, and *serA2* (intermediary metabolism and respiration); *Rv2814c* (insertion sequences and phages); and *mtp* (*Rv3312A*) (cell wall and cell processes) (Table 4).

Significant up-regulation between ∆*hbhA* and the WT was observed in genes involved in the following functional groups: *secE2* (cell wall and cell process); *fdxA,* and *csoR* (intermediary metabolism and respiration), *pfkB* (pentose phosphate pathway); *TB31.7* (virulence, detoxification and adaptation); *devS* and *devR* (regulatory proteins) and *Rv3128c* (conserved hypotheticals) (Table 3). A significant down regulation was noted in the following genes and their related pathways: *hbhA, mtp,* and *cfp2* (cell wall and cell process); *purA*, and *Rv0224c* (intermediary metabolism and respiration); *vapC5* and *Rv1082* (virulence, detoxification and adaptation); and *Rv2642c* (regulatory proteins) (Table 4).

Genes that displayed significant up-regulation between ∆*mtp-hbhA* and the WT were: *moaA1* and *lpdA* (intermediary metabolism and cell respiration); *ASdes* (conserved hypotheticals); and *PPE59* family (proline-glutamate and proline-proline-glutamate [PE/PPE]) (Table 3). Significant down-regulation was observed in: *mtp* and *hbhA* (cell wall and cell process); *Rv2042c* (conserved hypotheticals); *Rv3475*, *Rv2815c* and *Rv3326* (insertion sequences and phages) (Table 4).

Alterations in the central carbon metabolism (CCM), adenosine triphosphate (ATP) synthase, and cell wall transport and processes were observed in the absence of the adhesins. Significant up-regulation of the ATP synthase was observed in the absence of MTP, whilst significant alterations in cell wall and transport, virulence and regulation or proteins were observed in the absence of HBHA. In addition, genes associated with respiration and metabolism were up-regulated without MTP and HBHA.

#### Alterations in central carbon metabolism, ATP synthase, cell wall and cell processes in the absence of MTP and HBHA

**Fig. 1:**
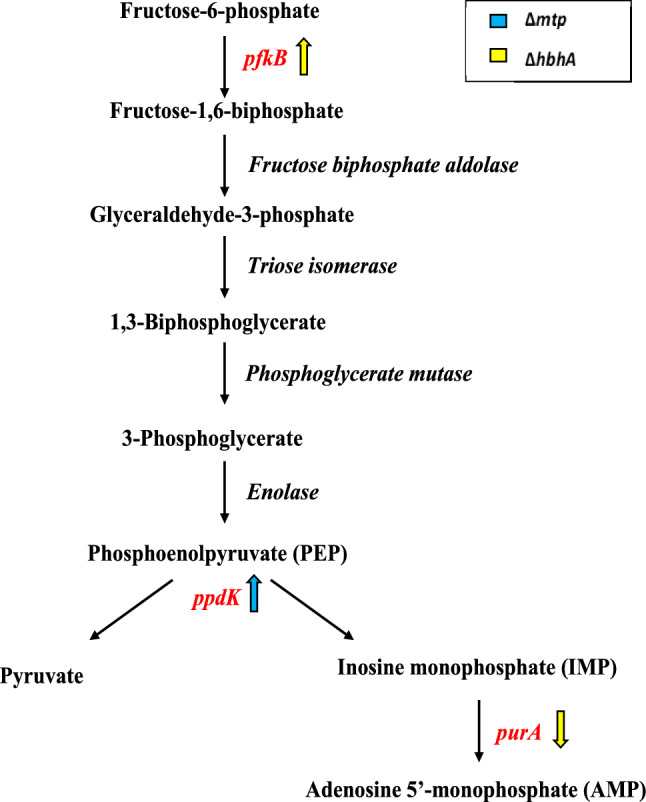
Transcriptomic analysis revealed alterations in the expression of genes involved in central carbon metabolism (CCM) in the *∆mtp* and *∆hbhA* strains. Significant up-regulation of *pfkB* and *ppdK* is involved in production of pyruvate during glycolysis. Down-regulation of *purA* indicates a decrease in AMP generation. Up- or down-regulation of genes is depicted by direction of the arrows

Alterations in expression levels of genes associated with CCM in the absence of MTP and HBHA might subsequently affect the production and utilization of ATP. The ∆*mtp* deletion mutant displayed significant up-regulation of *ppdK, icd1*, *ilvC* involved in CCM, and a significant down-regulation of genes associated with the biosynthesis of essential amino acids, tryptophan (*trpD*), and methionine (*mmuM*). Pyruvate phosphate dikinase (PPDK) is responsible for the reversible conversion of phosphoenolpyruvate (PEP) into pyruvate and adenosine 5’-monophosphate (AMP), or ATP (Sauer and Eikmanns 2005; Beste et al. 2013; Basu et al. 2018) (Fig. 1). The significant up-regulation of *ppdK,* may be associated with an increase in pyruvate production and AMP or an increase in ATP. Pyruvate is then converted to acetyl Coenzyme A (acetyl CoA), which enters the tricarboxylic cycle (TCA) (Sauer and Eikmanns 2005; Beste et al. 2013; Basu et al. 2018). Probable isocitrate dehydrogenase, *icd1*, is responsible for converting isocitrate into α-ketoglutarate and CO_2_ in the TCA cycle (Stryer et al. 1981; Banerjee et al. 2011). This rate-limiting step is the initial nicotinamide adenine dinucleotide hydrogen (NADH) yielding reaction of this cycle (Stryer et al. 1981; Banerjee et al. 2011). The increased expression of *icd1* may be associated with increased production of α-ketoglutarate, potentially resulting in an enhancement of the carbon flux through the TCA cycle. The ketol-acid reductoisomerase, encoded by *ilvC,* is involved in the biosynthesis of essential amino acids, valine and isoleucine, and is the second enzyme involved in the essential branched-chain amino acid (BCAA) biosynthesis (Armstrong and Wagner 1961; Amorim Franco and Blanchard 2017). Significant up-regulation of this second enzyme involved in the BCAA biosynthesis, may be associated with an increased production of 2,3-dihydroxy-3-methylbutanoate and 2,3-dihydroxy-3-methylpentanoate, precursors of valine and isoleucine (Armstrong and Wagner 1961; Amorim Franco and Blanchard 2017).

The ∆*hbhA* deletion mutant displayed significant up-regulation of *pfkB* and a substantial down-regulation of *purA* (Fig. 1). The up-regulation of *pfkB* is associated with increased production of glycolytic precursors, thereby enhancing of the carbon flux through the glycolysis pathway. The gene *purA* (Table 4 and Fig. 1), involved in the purine salvage pathway, encodes an adenylosuccinate synthase which is responsible for catalysing inosine monophosphate (IMP) to AMP (Stayton et al. 1983; Ducati et al. 2011; Kapopoulou et al. 2011). Significant down-regulation of *purA* suggests a decreased expression of genes associated with production of purines, essential compounds required for replication (Stayton et al. 1983; Ducati et al. 2011). This indicates that in the absence of HBHA, glycolysis is potentially enhanced, thus affecting ATP concentrations within the cell.

#### Alterations in differential gene expression of ATP synthase

**Fig. 2:**
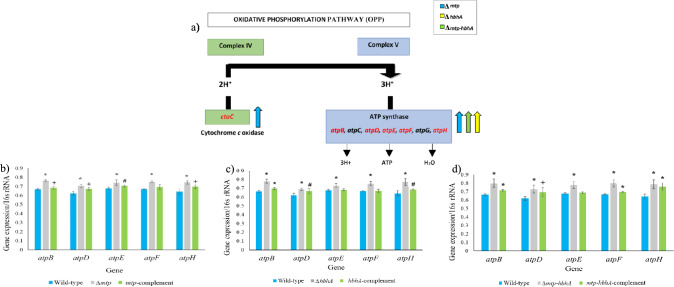
Transcriptomic analysis revealed alterations in the expression of genes involved oxidative phosphorylation (OPP) in the *∆mtp, ∆hbhA,* and *∆mtp-hbhA* strains. **a** Alterations in expression of genes associated with ATP synthase in *∆mtp, ∆hbhA*, and *∆mtp-hbhA* strains**.** Cytochrome *c* oxidase and ATP synthase involved in Complex IV and V during oxidative phosphorylation. Cytochrome *c* oxidase encoded by *ctaC* plays a role in complex IV (green) which feeds into complex V (blue) of the OPP. ATP is generated by ATP synthase encoded by *atpB, atpD, atpE, atpF, atpH* involved in complex V. Transcriptomic analysis revealed a significant up-regulation of *ctaC*, *atpE* and *atpF* in the ∆*mtp* deletion mutant, relative to WT. Up- or down-regulation of genes are depicted by direction of the arrows. **b–d** Absolute quantification method of interpretation (ThermoFisher Scientific 2022) was used to assess the RT-qPCR data. The gene expression levels were analysed relative to 16S rRNA for each gene of interest. Significance levels were established using an unpaired, parametric *t*-test for data between: **b** WT and *∆mtp,* and WT and *mtp*-complement; **c** WT and ∆*hbhA*, and WT and *hbhA-*complement; **d** WT and ∆*mtp-hbhA*, and WT and *mtp-hbhA-*complement. Between the WT and *∆mtp*, all genes displayed statistical significance: *atpB* (*p* < 0.001), *atpD* (*p* < 0.001), *atpE* (*p* = 0.002), *atpF* (*p* < 0.001), and *atpH* (*p* < 0.001). Between the WT and the *mtp*-complement, four of the genes displayed statistical significance; *atpB* (*p* = 0.049), *atpD* (*p* = 0.002), *atpE* (*p* = 0.004), *atpH* (*p* = 0.005). **c** Between the WT and ∆*hbhA*, five genes displayed statistical significance: *atpB* (*p* < 0.001), *atpD* (*p* < 0.001), *atpE* (*p* = 0.001), *atpF* (*p* < 0.001), and *atpH* (*p* < 0.001). Between the WT and the *hbhA*-complement, three gene displayed statistical significance: *atpB* (*p* < 0.001), *atpD* (*p* = 0.008), and *atpH* (*p* = 0.004). **d** Between the WT and *∆mtp-hbhA*, all genes displayed statistically significant differences: *atpB* (*p* < 0.001), *atpD* (*p* < 0.001), *atpE* (*p* < 0.001), *atpF* (*p* < 0.001), and *atpH* (*p* < 0.001). Between the WT and *mtp-hbhA-*complement, four genes displayed statistical significance: *atpB* (*p* < 0.001), *atpD* (*p* = 0.010), *atpF* (*p* < 0.001), and *atpH* (*p* < 0.001). Unpaired, parametric *t-*test relative to the WT: *, *p* < 0.001, #, *p* < 0.01, + ,* p* < 0.05

Significant alterations in expression of genes associated with the OPP was also observed. The OPP comprises five complexes, of which complex IV and V depicted significant up-regulation of the genes encoding the following: Cytochrome *c* oxidase (*ctaC*), ATP synthase (*atpB*, *atpD*, *atpE*, *atpF*) and ATP synthase transmembrane protein (*Rv1303*) encoding ATP synthase protein I (Table 3; Fig. 2a), in the ∆*mtp*. Cytochrome *c* oxidase encoded by *ctaC*, *ctaD* and *ctaE* plays a role in complex IV of OPP and feeds into complex V to generate ATP (Fig. 2a) (Sassetti et al. 2003; Rowland and Niederweis 2012). The mycobacterial F_0_-F_1_-ATP synthase is encoded by the *Rv1303-atpBEFGHDC-Rv1312* operon (Black et al. 2014). The *Rv1303* gene is located upstream of ATP synthase operon with a 7-bp overlap with *atpB* (Verma et al. 2014). The promoter for the ATP synthase operon is situated upstream of *Rv1303* and co-transcribes the genes encoding the different ATP synthase subunits (*atpB, atpC, atpD, atpE, atpF, atpH,* and *atpG*) (Verma et al. 2014). The RNA sequencing data revealed an overall significant up-regulation of the ATP synthase complex in ∆*mtp* (Tables 3 and S1) and non-significant up-regulation in ∆*hbhA*, and ∆*mtp-hbhA* (Tables S2 and S3), and cell wall transport and structural proteins (∆*mtp*, ∆*hbhA*, and ∆*mtp-hbhA*) (Tables 3 and 4 and S1–S3). Therefore, the sequencing data was used to compile a list of candidate genes (*atpB*, *atpD*, *atpE*, *atpF*, *atpH*, *lpqV*, *Rv0986*, *Rv0987*, and *secE2*) which were assessed by RT-qPCR using the WT, deletion mutants, and complemented strains*.*

The RT-qPCR results showed a similar trend to that observed in RNA sequencing for all five of the ATP synthase associated genes, thereby indicating validation of the latter expression data (Fig. 2b–d) [∆*mtp* (*p* ≤ 0.05)*,* ∆*hbhA* (*p* ≤ 0.05)*,* and ∆*mtp-hbhA* (*p* ≤ 0.05), relative to the WT]. Overall, all mutants induced a significantly higher level of gene expression (*p* ≤ 0.05) for all genes in comparison to their respective complemented and WT strains (Fig. 2b-d). Amongst the deletion mutants, ∆*mtp-hbhA,* induced the highest significant level of gene expression of *atpD* and *atpF* when compared to the WT (Fig. 2b–d). The expression means (Table S4) of the complements fall closer to the WT, than their individual deletion mutants for all genes. However, between the WT and *mtp-*complement, all genes displayed a significant increase in gene expression. Relative to the WT, the *hbhA*-complement and *mtp-hbhA*-complement displayed a significantly increased gene expression of three and four genes, respectively (Fig. 2b–d). The significant difference observed in all complement’s relative to the WT, could be attributed to the construction of the complements via non-integrating over-expressing plasmids.

#### Alterations in expression of genes associated with the cell wall and cell wall processes

**Fig. 3:**
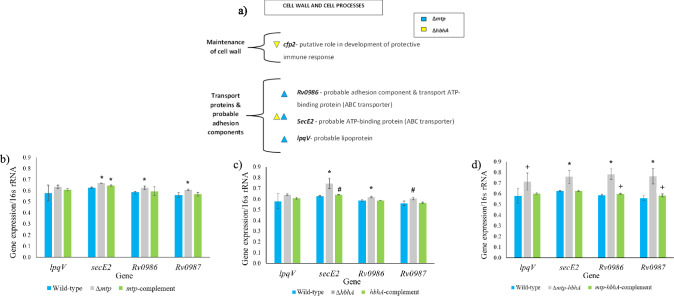
Transcriptomic analysis revealed alterations in the expression of genes involved in cell wall processes in the *∆mtp* and *∆hbhA* strains. **a** Alterations in gene regulation of cell wall maintenance, transport proteins and probable adhesion components. Significant down-regulation of *cfp2*, involved in the maintenance of the cell wall, was observed in the *∆hbhA* deletion mutant. Significant up-regulation of *lpqV,* encoding a possible membrane lipoprotein was demonstrated in *∆mtp*. *The* genes, *Rv0986*, and *secE2* form part of an ABC transporter. The gene *Rv0986* is a probable adhesion component and was significantly up-regulated in the *∆mtp* deletion mutant. The gene *secE2* was significantly up-regulated in the *∆mtp* and *∆hbhA* deletion mutants. Alterations in the *∆mtp*, *∆hbhA*, and *∆mtp-hbhA* deletion mutants relative to the WT are depicted by blue, yellow and green arrows, respectively. Up- or down-regulation of genes are depicted by direction of the arrows. **b–d** Reverse transcription quantitative PCR to assess expression of genes associated with adhesion molecules and ABC transporters (*secE2, Rv0986,* and *Rv0987*). Absolute quantification method of interpretation (ThermoFisher Scientific 2022) was used to assess the RT-qPCR data. The gene expression levels were analysed relative to 16S rRNA for each gene of interest. Significance levels were established using an unpaired, parametric *t*-test for data between: **b** WT and *∆mtp,* and WT and *mtp*-complement; **c** WT and ∆*hbhA*, and WT and *hbhA-*complement; **d** WT and ∆*mtp-hbhA*, and WT and *mtp-hbhA-*complement. **b** Between the WT and *∆mtp*, three genes displayed statistical significance *secE2* (*p* < 0.001), *Rv0986* (*p* < 0.001), and *Rv0987* (*p* < 0.001). Between the WT and the *mtp*-complement, one gene displayed statistical significance: *secE2* (*p* < 0.001). **c** Between the WT and ∆*hbhA*, three genes displayed statistical significance: *secE2* (*p* < 0.001), *Rv0986* (*p* < 0.001), and *Rv0987* (*p* = 0.001). Between the WT and the *hbhA*-complement, one gene displayed statistical significance: *secE2* (*p* < 0.010). (d) Between the WT and *∆mtp-hbhA*, all the genes displayed statistically significant differences: *lpqV* (*p* = 0.011), *secE2* (*p* < 0.001), *Rv0986* (*p* < 0.001), and *Rv0987* (*p* < 0.001). Between the WT and *mtp-hbhA-*complement, two genes displayed statistical significance: *Rv0986* (*p* = 0.020), and *Rv0987* (*p* = 0.040). Unpaired, parametric *t-*test relative to the WT: *, *p* < 0.001, #, *p* < 0.01, + ,* p* < 0.05

Apart from the perturbations in expression of genes associated with metabolism, alterations to regulation of genes associated with the cell wall/ cell wall-linked processes were observed in the absence of the adhesins. Transcriptomic analysis revealed a significant up-regulation of ABC transporter, *secE2*, probable adhesion component, *Rv0986,* and a probable exported protein, *Rv0988* (Pethe et al. 2004), in the ∆*mtp* (Table 3, Fig. 3a). The *Rv0986* gene forms part of the ABC transporter along with *Rv0987* and *secE2* (Daniel et al. 2018). Moreover, a non-significant up-regulation of *Rv0987* was observed in the ∆*mtp* (Table S1). The ∆*hbhA* displayed a significant up-regulation of *secE2* (Table 3). Furthermore, *Rv0986*, *Rv0987* and *secE2* showed a statistically non-significant up-regulation in the ∆*mtp-hbhA* (Table S3). Additionally, *lpqV*, encoding a possible membrane lipoprotein (Kapopoulou et al. 2011), was significantly up-regulated in the absence of MTP (Table 3). A significant up-regulation of *Rv3377c*, involved in the production of 1-tuberculosinyladenosine (1-TbAd), was observed in the ∆*mtp*. The terpene nucleoside is highly prevalent in mycobacterial strains and is involved in neutralizing acid pH of host cells vacuoles and causing a lysomotrophic effect on phagolysosomes, thereby aiding in the survival of intracellular mycobacteria during infection (Layre et al. 2014; Buter et al. 2019). The terpene nucleoside is a naturally abundant compound in *M. tuberculosis* (Layre et al. 2014; Buter et al. 2019). Significant down-regulation of *Rv2376c*, that encodes the putative secreted protein, CFP-2, was observed in the ∆*hbhA*. This protein is an early secreted component of the pathogen that may play a role in the protective immune responses (Webb et al. 1998). The significant down-regulation of this gene suggests alterations to this pathway. These findings suggest that the absence of the MTP and HBHA increases the expression of the above-mentioned ABC transporters to the cell wall.

RT-qPCR demonstrated a significant up-regulation of transport proteins and probable adhesion components in the absence of MTP and HBHA, as observed by the increased gene expression of *Rv0987* (*p* ≤ 0.05), *Rv0986* (*p* ≤ 0.05), and *secE2* (*p* ≤ 0.05) in all mutant strains, relative to the WT (Fig. 3b–d). The ∆*mtp* strain induced a significantly higher level of gene expression (*p* ≤ 0.05) for all genes, except *lpqV*, in comparison to the *mtp-*complement and WT (Fig. 3b–d). However, a large variance was observed in the expression of *lpqV* by the WT, possibly accounting for the non-significance between the WT and ∆*mtp* (Fig. 3b). The ∆*mtp-hbhA* induced the highest significant level of gene expression of *lpqV*, *Rv0986*, *Rv0987* in comparison to the WT (Fig. 3d). Relative to the WT, the *hbhA*-complement and *mtp-hbhA*-complement displayed a significantly increased gene expression of two genes (Fig. 3c, d). Between the WT and *mtp-*complement, one gene displayed a significant increase in gene expression (Fig. 3b). Significant differences between the complements and WT could be attributed to the over-expressing plasmid.

#### The bioluminescence assay phenotypically confirmed increased ATP utilization or lower ATP production in the absence of MTP and HBHA

**Fig. 4:**
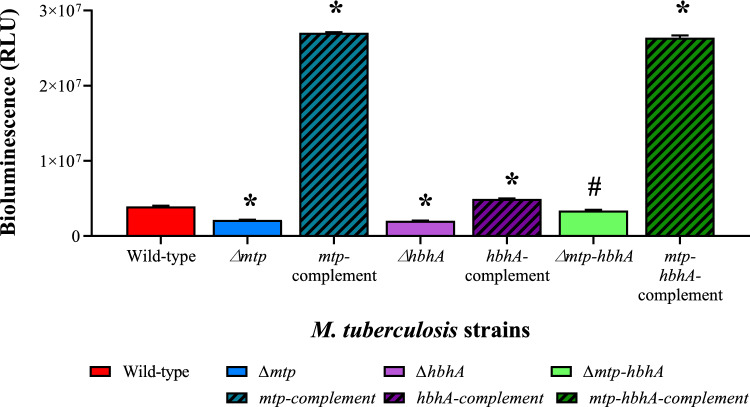
Bioluminescence assay depicting lower ATP levels in ∆*mtp,* ∆*hbhA*, ∆*mtp-hbhA* in comparison to *M. tuberculosis* V9124 WT. Significance levels were established using an unpaired, parametric *t-*test for comparison of WT to mutants and their respective complements. The results were significantly different between the mutants and wild type; ∆*mtp* (*p* ≤ 0.0001), ∆*hbhA* (*p* ≤ 0.0001), ∆*mtp-hbhA* (*p* = 0.0083) as well as between the complemented strains and WT; *mtp*-complement (*p* ≤ 0.0001), *hbhA*-complement (*p* ≤ 0.0001), *mtp*-*hbhA*-complement (*p* ≤ 0.0001). Unpaired, parametric *t-*test relative to the WT: *, *p* < 0.001, #, *p* < 0.01

The RNA sequencing and RT-qPCR revealed a similar trend of an overall increased expression of the ATP synthase in the absence of the adhesins. The bioluminescence assay revealed a significantly decreased concentration of ATP in the deletion mutants, relative to the WT (Fig. 4). Collectively, this is suggestive of a higher ATP consumption in the mutants as a compensatory response to the alternate metabolic or cellular processes induced by the absence of the adhesin/s. Potential metabolic or cellular pathways enhanced by the absence of the adhesin/s include ATP-dependant ABC transporters and adhesion molecules. Alternately, this may reflect a lower ATP production via alternative pathways, such as a reduced proton gradient due to the absence of adhesins (Rao et al. 2008; Black et al. 2014). The significant differences in the ATP concentrations in the WT and complemented strains could be attributed to the construction of the complements via non-integrating over-expressing plasmids. Additionally, if the deletion of the adhesins potentially resulted in a reduced proton motive force (PMF), it is postulated that the over-expressing complements, viz., the non-integrating pMV261 plasmids, will potentially increase the PMF, thereby, explaining the increased concentration of ATP in the complements.

## Discussion

The current study demonstrated the modulatory role of MTP and HBHA, individually and in combination, on the transcriptome of *M. tuberculosis*. The ∆*mtp*, ∆*hbhA* and ∆*mtp-hbhA,* showed an overall increased expression of ATP synthase suggesting that *M. tuberculosis* attempts to increase ATP generation via OPP in the absence of MTP, HBHA and MTP-HBHA. The preference toward this pathway for ATP generation may be attributed to alterations in PMF caused by the absence of the surface located adhesins. Furthermore, reduced concentrations of ATP in the three deletion mutant strains*,* suggest either increased ATP consumption or decreased ATP generation via PMF. Additionally, an up-regulation of genes associated with ATP-dependant ABC cell membrane transport proteins in the ∆*mtp*, ∆*hbhA* and ∆*mtp-hbhA,* suggest increased ATP utilization for cell wall transport. The absence of MTP was associated with an up-regulation of genes associated with lipoprotein biosynthesis, potentially increasing membrane lipoproteins to enhance lipid attachment and immune response to accommodate for the lack of the adhesin. The absence of MTP-HBHA was associated with the pathogen favouring carbohydrate metabolism by up-regulating glycolysis associated genes in attempts to compensate for lower lipid metabolism through the TCA cycle. Collectively, the combined absence of both adhesins, was associated with a greater impact on cell wall biosynthesis, ATP consumption, and cell wall transport in comparison to the absence of the adhesins individually.

### 4The absence of MTP and HBHA potentially enhances expression of genes associated with cell wall lipid and lipoprotein biosynthesis, and reduces the production of genes associated with proinflammatory molecules in recipient macrophages

The significant up-regulation of *lpqV* (*Rv1064c*) in ∆*mtp*, *ASdes* in ∆*mtp-hbhA*, and significant down regulation of *cfp2* (*Rv2376c*) in ∆*hbhA* (Table 4; Fig. 3a), suggested alterations in expression of genes involved in various cell wall associated pathways in the deletion mutants relative to the WT.

The 19kD putative membrane lipoprotein, *lpqV,* was hypothesized to be a probable toll-like receptor 2 (TLR-2) ligand and plays a role in the development of the immune response (Gehring et al. 2004). In the present study, RT-qPCR revealed a significantly increased expression of *lpqV* in the ∆*mtp-hbhA*, while ∆*mtp* and ∆*hbhA* displayed a non-significant increased expression, relative to the WT. The non-significant increased expression of *lpqV* in the ∆*mtp* and ∆*hbhA,* could be attributed to the large variance in the expression of *lpqV* by the WT. The significant up-regulation of *lpqV*, indicated by the RNA sequencing, and the non-significant increased expression of *lpqV*, indicated by the RT-qPCR in ∆*mtp*, suggests that the absence of MTP is associated with alterations in the synthesis of a membrane protein to potentially enhance lipid attachment and immune response as a compensatory mechanism. All the complemented strains displayed no significant differential expression of *lpqV* relative to the WT, indicating that they were restored to the WT genotype. The differential expression of *lpqV in* ∆*hbhA* and ∆*mtp-hbhA* quantified by RNA sequencing varies from the gene expression quantified by RT-qPCR. Thus, the differing expression cannot be used to deduce a significant role of *lpqV* in the ∆*hbhA* and ∆*mtp-hbhA*. Apart from this*,* majority of the selected genes showed a similar trend in expression in both techniques. Therefore, both techniques are reliable and accurate methods of detecting changes in gene expression.

The small regulatory RNA, *ASdes*, is involved in mycolic acid biosynthesis and postulated to play a role in regulation of lipid metabolism (Singh et al. 2016). Significant up-regulation of *ASdes* in the ∆*mtp-hbhA* indicates that the simultaneous absence of both MTP and HBHA, is associated with a potential increase in lipid biosynthesis in the cell membrane. The putative early secreted protein CFP-2, encoded by *cfp2* (*Rv2376c*), of the pathogen, plays a vital role in proinflammatory responses through mitogen-activated protein kinase (MAPK) pathway during the early stages of infection in humans (Webb et al. 1998, Lee et al. 2006). However, the T-cell immunoreactivity of the CFP-2 protein is weaker in comparison to the 30-kDa antigen (Lee et al. 2006). The significant down-regulation of this gene in ∆*hbhA* is associated with a down-regulation of CFP-2 pathogen-associated molecular patterns (PAMPs), which stimulate the production of proinflammatory molecules in recipient macrophages (Lee et al. 2006, Bhatnagar and Schorey 2007; Smith et al. 2017; Wang et al. 2019). The deletion of *mtp* increases expression of genes associated with lipoprotein biosynthesis, potentially increasing membrane lipoproteins, lipid attachment and immune response, to accommodate for the lack of the adhesin.

### MTP and HBHA modulate energy production in *M. tuberculosis* by regulating expression of genes associated with CCM

The CCM is defined as the enzymatic repurposing of carbon substrates through various pathways which include: gluconeogenesis, TCA cycle, glycolysis and the pentose phosphate shunt (Rhee et al. 2011). In the present study, alterations in several pathways of CCM were observed in the absence of the adhesins.

#### MTP and HBHA influence expression of genes associated with gluconeogenesis/glycolysis pathways linked to the TCA cycle

Fructose-6-phosphate, by way of fructose-1,6-biphosphate, is converted into either glyceraldehyde-3-phosphate (which undergoes glycolysis/gluconeogenesis), or dihydroxyacetone phosphate (which utilizes NADH) to produce glycerol-3-phosphate (G3P)) (Kapopoulou et al. 2011; Phong et al. 2013). Since *pfkB* is the enzyme responsible for the rate determining step of the glycolysis pathway (Kapopoulou et al. 2011; Phong et al. 2013), the significant up-regulation of this gene in the absence of HBHA (Fig. 1; Table 3), indicates an increased expression of glycolytic precursors, potentially resulting in an enhancement in the carbon flux, increasing production of pyruvate. Adenylosuccinate synthase (ADSS), encoded by *purA*, is responsible for the first committed step in the conversion of IMP to AMP in the purine salvage pathway (Stayton et al. 1983; Ducati et al. 2011; Kapopoulou et al. 2011). The significant down-regulation of *purA* in the ∆*hbhA* (Fig. 1; Table 4), is associated with a reduced need for purine synthesis.

Pyruvate phosphate dikinase (PPDK), is the enzyme that drives both gluconeogenesis and glycolysis (Kayne 1973; Noy et al. 2016). Under conditions of low oxygen levels, PPDK drives the glycolysis pathway towards pyruvate production with the release of ATP, and under conditions of higher oxygen levels, PPDK drives the gluconeogenesis pathway towards PEP production with the release of adenosine 5’-monophosphate (AMP) (Kayne 1973; Noy et al. 2016). Apart from PPDK, pyruvate kinase (PK) is one of the rate-limiting steps of glycolysis, therefore, potentially controlling the flux through and out of glycolysis (Kayne 1973; Noy et al. 2016). Pyruvate and PEP can either serve as precursors for anabolism or enter the TCA cycle via acetyl Co-A (Sauer and Eikmanns 2005; Beste et al. 2013; Basu et al. 2018). The significant up-regulation of *ppdK*, along with the up-regulation of genes involved in the TCA cycle, potentially point toward the pathogen enhancing glycolysis, in the absence of *mtp*. This may suggest the pathogen up-regulating genes associated with pathways linked to carbohydrate consumption. Thus, MTP and HBHA, individually or in combination, influence the expression of genes associated with the TCA cycle aiding in the pathogen’s replication.

#### MTP influences expression of genes associated with the TCA cycle

Significant alterations in the expression of genes associated with the TCA cycle was observed in ∆*mtp*. These include the down-regulation of *serA2* and *mmuM*, and the up-regulation of *ilvC* and *icd1*. Pyruvate enters the TCA cycle for energy generation (Sauer and Eikmanns 2005; Beste et al. 2013; Basu et al. 2018), and is involved in the generation of serine, an essential amino acid for bacterial growth (Chattopadhyay et al. 2007; Ågren et al. 2008). Synthesis of 3-phosphoglycerate, precursors of L-serine, is catalysed by *serA2* (*Rv0728c*), and *serC* (Chattopadhyay et al. 2007; Ågren et al. 2008). The deletion of the MTP adhesin in the present study, was associated with alterations in metabolism that slow down replication, potentially decreasing the requirement of proteins, thereby decreasing expression of genes responsible for synthesis of amino acids like serine, from the intermediates of carbohydrate or lipid metabolism.

Microorganisms produce signalling molecules called alarmones (Hauryliuk et al. 2015; Fang and Bauer 2018), which are regulated by alarmone synthetase/hydrolase enzymes (Shivers and Sonenshein 2004; Hauryliuk et al. 2015). An example of these signalling molecules are the Rel proteins found in microorganisms such as *Eschericha coli, Rhodobacter capsulatus* (Shivers and Sonenshein 2004; Hauryliuk et al. 2015), and *M. tuberculosis* (RelA) (Avarbock et al. 2000; Ronneau and Hallez 2019). This enzyme contains an ACT domain that plays a probable role in controlling amino acid metabolism (Shivers and Sonenshein 2004; Hauryliuk et al. 2015). A study on *Rhodobacter capsulatus* reported that the ACT domain in the Rel alarmone synthase/hydrolase binds to BCAAs, valine and isoleucine, the cellular concentrations of which directly affect the alarmone activity (Fang and Bauer 2018). An increased expression of the ketol-acid reductoisomerase (*ilvC*) observed in the current study, suggests an increase in precursors involved in the biosynthesis of the essential branched-chain amino acids (BCAAs), valine and isoleucine (Armstrong and Wagner 1961; Amorim Franco and Blanchard 2017). Thus, in the current study, in the absence of *mtp*, the significant up-regulation of the BCAAs is associated with increased valine and isoleucine biosynthesis, potentially increasing stimulation of the alarmone, which may signal that the pathogen is in stress.

Isocitrate dehydrogenase (*icd1*) catalyses a vital rate-limiting step in the TCA cycle (Stryer et al. 1981; Banerjee et al. 2011). Significant up-regulation of *icd1* indicates an increase in the flux through this pathway. *M. tuberculosis* has a B12-independent methionine synthase responsible for the synthesis of L-methionine from L-homocysteine. Methionine is a precursor for succinyl-coA, a component of the TCA cycle (Warner et al. 2007). Synthesis of L-methionine can also be catalysed by *mmuM*, a homocysteine *S*-methyltransferase (Warner et al. 2007). Studies have reported that *mmuM* is absent in rapid growing mycobacteria, however, it is conserved in the reduced genome of *M. leprae* (Pejchal and Ludwig 2004; Young et al. 2015). Since L-methionine initiates the synthesis of most proteins (Pejchal and Ludwig 2004; Warner et al. 2007; Young et al. 2015), the significant down-regulation of *mmuM* in the current study, may be associated with a decreased expression of L-methionine linked proteins, potentially decreasing replication of *M. tuberculosis* in the absence of the adhesin. Therefore, MTP influences the expression of genes associated with alarmone production, serine and methionine biosynthesis in the TCA cycle, contributing to the virulence of *M. tuberculosis.*

#### MTP and HBHA modulate expression of genes associated with ATP synthesis via the OPP pathway

During OPP, ATP is generated via the F_1_F_0_-ATP synthase enzyme, coupled to the PMF (Butlin et al. 1971; Black et al. 2014). *M. tuberculosis*, an obligate aerobe, is dependent on OPP for growth and survival during pathogenesis (Butlin et al. 1971; Black et al. 2014). The mycobacterial F_1_-F_0_-ATP synthase is encoded by the *Rv1303-atpBEFGHDC-Rv1312* operon (Black et al. 2014). The PMF is established through the development of the transmembrane proton gradient, which occurs when electrons move through the electron transport chain resulting in the establishment of membrane potential (Rao and Ranganathan 2004; Black et al. 2014). The electron transport chain and PMF are vital components for the generation of ATP, a crucial requirement for metabolic processes. The OPP is the predominant source of energy production in mycobacteria and ATP synthase represents the pivotal enzyme in ATP generation in mycobacteria (Black et al. 2014). *M. tuberculosis* is known to survive at low PMF (−110 mV) (Rao and Ranganathan 2004), and the ATP synthase needs to be adapted to allow efficient energy usage at this low PMF for ATP synthesis (Lu et al. 2014). In the present study, with the increased expression of the ATP synthase associated with the deletion of MTP and HBHA, it is postulated that the proton gradient may be altered in the ∆*mtp*, ∆*hbhA*, and ∆*mtp-hbhA* deletion mutant strains, potentially resulting in a decreased ATP production via this pathway. Subsequently, this decrease may potentially signal attempts to increase ATP synthesis via ATP synthase.

The synthesis of ATP via OPP is fed by cytochrome *c* oxidase (*ctaC*) located in Complex IV, (Shi et al. 2005; Rowland and Niederweis 2012), and is largely mediated by ATP synthase of Complex V (Iino and Noji 2013) (Fig. 2a). In the current study, RNA sequencing data demonstrated perturbations of genes involved in Complex IV (*ctaC*) and V (*atpE* and *atpF)* and transmembrane protein encoding ATP synthase protein I (*Rv1303*) of the OPP (Table 3; Fig. 2a. These genes were significantly up-regulated in ∆*mtp*, relative to the WT, suggesting an attempt to increase synthesis of ATP via this pathway under low PMF conditions created by the adhesin depletions. In addition, bioinformatic analysis showed alterations in complex V in ∆*mtp-hbhA* (statistically non-significant up-regulation of *atpB*, *atpC* and *atpH*) (Table S3) and ∆*hbhA* (statistically non-significant up-regulation of *atpB*) (Table S2).RT-qPCR analysis of *atpB, atpD, atpE, atpF,* and *atpH* demonstrated an increased expression of ATP synthase in all three deletion mutants, suggesting attempted increase in ATP generation via the OPP pathway in the absence of the adhesins. Between the WT and complemented strains, few genes displayed statistical significance suggesting that the latter were only partially restored to the WT genotype. This significant difference could be attributed to the construction of the complements via non-integrating over-expressing plasmids.

Despite the increased ATP synthase gene expression, the functional ATP assay showed a significantly decreased concentration of ATP in the deletion mutants, relative to the WT. It is speculated that the decreased concentration of ATP in the deletion mutants could be attributed to alterations to the proton gradient. Alternatively, the decreased concentration of ATP could be the result of an increased ATP consumption via ATP-dependant ABC transporters and adhesion molecules. A significantly increased concentration of ATP was observed in the complements, relative to the WT. This could be attributed to either a higher energy storage observed in the complements, particularly the ∆*mtp-* and ∆*mtp-hbhA-*complements, relative to the WT, or an increase in ATP generation via PMF. Collectively, the data suggests that the absence of MTP and HBHA perturbs ATP synthase, potentially increasing ATP synthesis, which may be signalled by alterations to the proton gradient.

Moreover, significant up-regulation of *lpdA* (*Rv3303c*), a lipoamide dehydrogenase (Akhtar et al. 2006), was evident in Δ*mtp-hbhA*. The *lpdA* gene is predicted to manage oxidative stress in pathogenic bacteria (Argyrou and Blanchard 2001; Argyrou et al. 2004; Leung et al. 2017). Although the effect of *lpdA* on drug resistance in *M. tuberculosis* remains unknown, its involvement in regulation of the redox equilibrium suggests a potential association with drugs that require bacterial catalase activation (Leung et al. 2017). Taken together, these results indicate that MTP and HBHA influence the expression of genes associated with regulation of OPP in *M. tuberculosis.* The influence of MTP and HBHA on OPP may impact the ability of *M. tuberculosis* to successfully infect and proliferate within the host and may alters the pathogens’ ability to exhibit antibiotic resistance to OPP targeting drugs, such as bedaquiline. Further research in vitro or in vivo infection models is needed to verify this.

### The role of MTP and HBHA in transport across the cell wall and cell membrane

*M. tuberculosis* has numerous cell wall transport proteins that aid in its survival and persistence (Youm and Saier Jr 2012). In the current study, RNA sequencing analysis revealed alterations to the expression of various genes encoding transport proteins and operons, suggesting an increase in cell wall transport in the absence of the adhesins. The *Rv0986, Rv0988*, and *secE2* (*Rv0379*) in ∆*mtp*, and *secE2* in ∆*hbhA* were significantly up-regulated (Tables 3 and 4; Fig. 3b–d). In *M. tuberculosis*, *Rv0986*, *Rv0987* and *secE2* encode an ATP-binding cassette (ABC) transporters which are involved in energy coupling to the transport system (Pethe et al. 2004; Rosas-Magallanes et al. 2006). The ABC transporters require ATP for transport of adhesion molecules, such as *Rv0987*, across the membrane and host cell binding, and are dependent on energy production in the cell (Kuroda and Tsuchiya 2009; Black et al. 2014). Moreover, *secE2* (*Rv0379*), encodes a possible ABC transport protein of the subfamily F (Kapopoulou et al. 2011) and displays strong ATPase activity (Daniel et al. 2018). RT-qPCR analysis of *Rv0986, Rv0987* and *secE2* of the ATP-binding cassette (ABC) transporters demonstrated that expression of these genes was significantly increased in ∆*mtp,* ∆*hbhA* and ∆*mtp-hbhA*, relative to the WT, following a similar trend compared to the RNA sequencing. The *mtp-* and *hbhA-*complemented strains displayed no significant expression of *Rv0986* and *Rv0987* relative to the WT, whilst the *mtp-hbhA-*complement displayed no significant expression of *secE2* relative to the WT. Overall up-regulation of these genes are associated with increased transport of proteins to the cell membrane/wall via ATP utilization in the absence of MTP and HBHA. Additionally, *Rv0988,* a probable exported protein (Pethe et al. 2004; Rosas-Magallanes et al. 2006) was significantly up-regulated in ∆*mtp*, suggesting alterations in membrane associated proteins in the absence of the pili.

The deletion of the genes individually, or in combination, did not significantly alter the bacterial transcriptome. Therefore, the relative paucity of SDEGs observed is expected. *M. tuberculosis* possesses a multitude of adhesins (Pethe et al 2001; Krachler and Orth 2013; Kumar et al 2013), as well as other molecules that perform “moonlighting” functions (Cehovin et al. 2010; Henderson and Martin 2011; Boradia et al. 2014), that most likely compensate for the absence of others. Furthermore, the possibility of the adhesins exerting post-translational modifications may explain the low number of SDEGs (Zhong et al. 2023). A limitation of the present study is the exclusion of infection models. Since the impact of MTP and HBHA was elucidated in a bacterial transcriptome model, the findings cannot be directly extrapolated to an infection model. Future studies could investigate in vitro models to further understand how the effect on the deletion mutants during different infection phases. The lack of proteomic validation is another limiting factor, which could be investigated in future studies that include infection models. In addition, a comparative analysis amongst the mutant strains was not performed. Future investigations could perform a more detailed comparative analysis of the mutant strains to determine whether these adhesins coregulate these common pathways. The significant differences between the complemented strains and WT indicate incomplete restoration. However, the authors believe that since the trend of the complemented strains were the reverse of the gene deletion mutants, it indicates that the gene function was partially restored.  Therefore, complementation with an integrative vector should be considered for future work. In addition, bioenergetic studies could provide the percentage of ATP generated by each cellular pathway (PMF, OPP, glycolysis) in order to determine alterations in ATP generation. Moreover, only the alterations in expression of the ATP synthase genes were further investigated via RT-qPCR and a bioluminescence assay. Future studies could investigate growth kinetics or cell wall composition analysis to provide direct experimental validation of the transcriptional changes observed. This study was limited to one clinical strain which may not be representative of other clinical strains. Despite the above limitations, this study provides a novel and valuable insight on the roles that MTP and HBHA play in the regulation of the *M. tuberculosis* transcriptome.

## Conclusion

RNA sequencing analysis coupled with gene expression and a bioluminescence assay revealed that deletion of the *mtp* and *hbhA* genes resulted in perturbations to the *M. tuberculosis* transcriptome*.* The major alterations were associated with respiration and metabolism, cell wall and cell processes. An overall increased expression of ATP synthase was observed in ∆*mtp*, ∆*hbhA* and ∆*mtp-hbhA,* suggesting attempts of the adhesin depleted mutant strains toward increasing the generation of ATP via this pathway. The increased ATP generation indicated by the increased expression the ATP synthase, could be attributed to alterations in PMF, resulting from a perturbed proton gradient due to the lack of surface adhesins. The bioluminescence assay displayed reduced concentrations of ATP in all mutants, relative to the WT, suggestive of either increased ATP consumption or decreased ATP generation via PMF. The observed perturbations indicate that MTP and HBHA influence the expression of genes associated with the TCA cycle, cell wall/cell wall transport, OPP, and alarmone production, thereby aiding in the survival and replication of *M. tuberculosis*. Moreover, the deletion of both MTP and HBHA displayed a greater impact on the mentioned pathways in comparison to the single deletion of each adhesin gene. Therefore, this study further supports the use of MTP and HBHA in combination as an important target for TB diagnostics and therapeutic interventions. Furthermore, the pathway genes, involved in OPP, amino acid synthesis, cell wall associated transport and processes, associated with these two adhesins have been identified as potential novel targets for this purpose.

## Supplementary Information

Below is the link to the electronic supplementary material.

Supplementary file1 (DOCX 166 KB)

## Data Availability

Data is provided within the manuscript or supplementary information files.

## Abbreviations

*M. tuberculosis*: *Mycobacterium tuberculosis*
MTP: *Mycobacterium tuberculosis* Curli pili
HBHA: Heparin-binding hemagglutinin adhesin
WT: Wild-type
*∆mtp*: *mtp-*Deletion mutant
*∆hbhA*: *hbhA-*Deletion mutant
*∆mtp-hbhA*: *mtp-hbhA-*Deletion mutant
TB: Tuberculosis
HIV: Human immunodeficiency virus
ATP: Adenosine triphosphate
PMF: Proton motive force
MDR/XDR-TB: Multi-drug resistant tuberculosis or extensive drug resistant tuberculosis
GTC: Guanidine thiocyanate
GTF: Gene transfer format
FPKM: Fragments per kilobase of transcript per million
FC: Fold-change
KEGG: Kyoto encyclopaedia of genes and genomes
OADC: Oleic albumin dextrose catalase
OD: Optical density
CCM: Central carbon metabolism
acetyl CoA: Acetyl coenzyme A
PPP: Pentose phosphate pathway
TCA: Tricarboxylic acid
AMP: 5’-Monophosphate
PEP: Phosphoenolpyruvate
PPDK: Pyruvate phosphate dikinase
IMP: Inosine monophosphate
OPP: Oxidative phosphorylation pathway
BCA: Branched chain amino acid
BCG: Bacille Calmette-Guérin’
RT-qPCR: Real time reverse transcription quantitative polymerase chain reaction
PCR: Polymerase chain reaction
MAPK: Mitogen activated protein kinase
G3P: Glycerol-3-phosphate
ADSS: Adenylosuccinate synthase
ABC: Adenosine triphosphate binding cassette
