# Supplementary material for: Mycobacterium tuberculosis curli pili (MTP) and heparin-binding hemagglutinin adhesin (HBHA) facilitate regulation of central carbon metabolism, enhancement of ATP synthesis and cell wall biosynthesis
Source: Arch Microbiol. 2025 May 28;207(7):156. doi: 10.1007/s00203-025-04352-w (PMC12119724; doi:10.1007/s00203-025-04352-w)
Supplement: Supplementary file 1 — Supplementary file1 (DOCX 166 KB) [file 203_2025_4352_MOESM1_ESM.docx]

# Supplementary Information (SI)

**Strain confirmation**


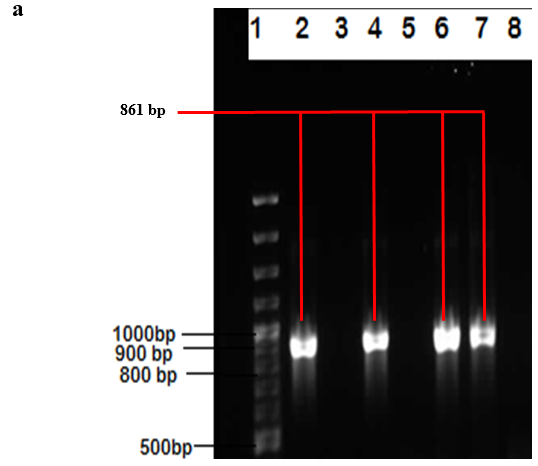

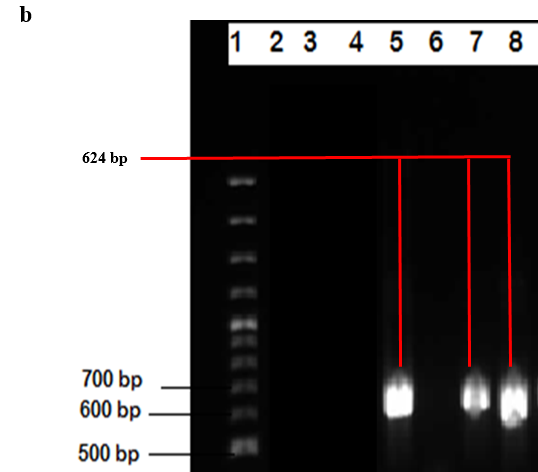


**Figure S1. Gel electrophoresis images of PCR products confirming bacterial strains run at 70V for 3 hours on a 1.5% agarose gel using 100 bp (BioLabs) marker.** (a) PCR targeting the *mtp* gene. (Lane 1) Molecular weight marker, (Lane 2) wild-type (V9124), (Lane 3) *∆mtp*, (Lane 4) *mtp*-complement, (Lane 5) *∆mtp-hbhA*, (Lane 6) *mtp*-*hbhA*-complement, (Lane 7) *mtp*-*hbhA*-complement, (Lane 8) negative control.(b) PCR targeting the *hbhA* gene. (Lane 1) Molecular weight marker, (Lane 2) negative control, (Lane 4) *∆hbhA*, (Lane 5) wild-type (V9124), (Lane 6) *∆mtp-hbhA*, (Lane 7) *hbhA* -complement, (Lane 8) *mtp*-*hbhA*-complement.

| **Table S1.** Differentially expressed up- and down-regulated genes induced by the Δ*mtp* deletion mutant relative to the wild-type. | | | | | | | | | |
| --- | --- | --- | --- | --- | --- | --- | --- | --- | --- |
| **Gene**  **Table C2.2** Differentially expressed up- and down-regulate genes induced by the Δ*mtp* deletion mutants relative to the wild-type. | **Gene ID** | **FPKM Δ*mtp1*** | **FPKM Δ*mtp2*** | **FPKM Δ*mtp3*** | **FPKM WT1** | **FPKM WT2** | **FPKM WT3** | **FC** | ***p-value*** |
| **Up-regulated genes** | | | | | | | | | |
| *ilvC* | MSTRG.  1993 | 174.3111 | 151.8738 | 181.4549 | 103.8889 | 144.3416 | 110.0690 | 1.3181 | 0.0002 |
| *PE_PGRS62* | MSTRG.  2517 | 297.2071 | 238.2724 | 334.0728 | 168.8085 | 262.5674 | 166.8093 | 1.3049 | 0.0050 |
| *Rv3126c* | MSTRG.  2055 | 238.3167 | 219.9807 | 263.7231 | 133.4774 | 223.3391 | 142.6992 | 1.3199 | 0.0050 |
| *Rv1303* | MSTRG.896 | 176.8377 | 182.0453 | 202.7130 | 116.4416 | 149.6191 | 121.8141 | 1.3747 | 0.0057 |
| *icd1* | MSTRG.2194 | 200.7752 | 163.3776 | 198.0125 | 120.9552 | 162.1805 | 117.4838 | 1.3006 | 0.0057 |
| *Rv2477c* | MSTRG.1678 | 6.7454 | 3.2804 | 0.0334 | 1.5333 | 0.0582 | 1.1044 | 2.3400 | 0.2880 |
| *Rv0987* | MSTR G.691 | 15.3529 | 9.8796 | 15.2727 | 4.2557 | 5.4195 | 9.1302 | 1.9900 | 0.9400 |
| *metE* | MSTRG.786 | 3.9581 | 2.3741 | 1.6625 | 1.6233 | 0.2936 | 2.2479 | 1.8888 | 0.0968 |
| *gap* | MSTRG.958 | 2.2144 | 2.0641 | 0.0047 | 1.9951 | 0.0135 | 0.0628 | 1.7910 | 0.3359 |
| *tesA* | MSTRG.1937 | 0.7650 | 1.1654 | 1.4550 | 0.8264 | 0.0441 | 0.6384 | 1.5940 | 0.0658 |
| *secE2* | MSTRG.  2681 | 177.9619 | 144.4083 | 177.8106 | 89.0626 | 148.2808 | 84.5948 | 1.4030 | 0.0058 |
| *ctaC* | MSTRG.1461 | 20.0976 | 22.2502 | 18.8812 | 11.2714 | 11.2156 | 13.6725 | 1.6900 | 0.0088 |
| *PPE56* | MSTRG.2197 | 24.8621 | 17.2419 | 25.0241 | 1.6904 | 2.1092 | 4.5244 | 6.4249 | 0.0088 |
| *atpE* | MSTRG.897 | 378.2178 | 388.4163 | 394.9370 | 286.2736 | 312.7594 | 258.8344 | 1.3201 | 0.0100 |
| *PPE67* | MSTRG.2448 | 215.9785 | 197.4763 | 248.0787 | 127.0865 | 182.1322 | 146.5528 | 1.3474 | 0.0101 |
| *lpqV* | MSTRG.753 | 2.2635 | 0.9019 | 2.7531 | 0.0234 | 0.8229 | 0.0167 | 1.9278 | 0.0141 |
| *Rv1928c* | MSTRG.1287 | 0.9066 | 0.6104 | 0.8719 | 0.2040 | 0.3905 | 0.3577 | 1.3173 | 0.0192 |
| *ppdK* | MSTRG.2179 | 1.1435 | 0.8546 | 1.1799 | 0.6021 | 0.1497 | 0.3920 | 1.3746 | 0.0256 |
| *Rv3377c* | MSTRG.783 | 63.9615 | 74.9208 | 67.1147 | 44.4408 | 55.0552 | 44.2110 | 1.5623 | 0.0281 |
| *Rv0988* | MSTRG.2212 | 1.9429 | 1.5008 | 1.4895 | 0.5832 | 0.5805 | 1.0835 | 1.3843 | 0.0325 |
| *atpF* | MSTRG.691 | 485.9854 | 413.2196 | 434.4684 | 338.9169 | 315.4052 | 264.3184 | 1.5522 | 0.0391 |
| *Rv3857c* | MSTRG.898 | 261.4347 | 255.8223 | 345.8023 | 179.2441 | 237.9914 | 182.8822 | 1.3817 | 0.0433 |
| *PE25* | MSTRG.2561 | 571.4044 | 418.9909 | 465.5733 | 311.3480 | 376.6409 | 304.1723 | 3.8476 | 0.0441 |
| *SecE2* | MSTRG. 281 | 177.9619 | 144.4083 | 177.8106 | 89.0626 | 148.2808 | 84.5948 | 1.4031 | 0.0058 |
| *atpB* | MSTRG. 896 | 460.2688 | 484.7766 | 396.3477 | 275.7222 | 347.5646 | 243.7619 | 1.4926 | 0.0496 |
| *atpH* | MSTRG. 899 | 417.9018 | 366.2022 | 372.9645 | 294.8143 | 274.8796 | 175.9374 | 1.5308 | 0.1142 |
| *atpA* | MSTRG. 900 | 286.2702 | 246.4708 | 263.2965 | 214.0836 | 216.2528 | 175.4323 | 1.2802 | 0.0617 |
| *atpG* | MSTRG. 901 | 273.5947 | 264.8813 | 248.6554 | 215.4467 | 199.9504 | 134.0745 | 1.4265 | 0.1389 |
| *atpD* | MSTRG. 902 | 245.5496 | 225.5711 | 243.0976 | 189.2383 | 185.6675 | 136.7313 | 1.3636 | 0.0798 |
| *atpC* | MSTRG. 903 | 217.9742 | 222.4428 | 256.6363 | 202.9863 | 201.9573 | 128.5401 | 1.2669 | 0.2662 |
| **Down-regulated genes** | | | | | | | | | |
| *mtp* | MSTRG.2175 | 61.1317 | 64.6229 | 69.5307 | 117.9687 | 212.2222 | 110.8945 | 0.4102 | 0.0040 |
| *Rv2814c* | MSTRG.1871 | 2.6333 | 0.7248 | 2.8509 | 1.5099 | 6.1063 | 2.6119 | 0.5584 | 0.0414 |
| *serA2* | MSTRG.511 | 79.7609 | 64.5728 | 85.2790 | 77.2353 | 116.3066 | 97.0046 | 0.7311 | 0.0277 |
| *trpD* | MSTRG.1461 | 2.2069 | 1.8813 | 2.7136 | 2.6994 | 4.1166 | 2.5272 | 0.7314 | 0.0054 |
| *ruvA* | MSTRG.1747 | 112.8308 | 74.6945 | 114.8190 | 98.7666 | 156.6116 | 106.9883 | 0.7382 | 0.0372 |
| *mmuM* | MSTRG.1662 | 107.4678 | 81.1709 | 134.2105 | 99.4332 | 174.6356 | 106.8598 | 0.7392 | 0.0221 |
| *moeX* | MSTRG.1123 | 83.7515 | 80.0692 | 105.3587 | 96.5335 | 146.6090 | 88.4474 | 0.7398 | 0.0272 |
| *Rv2026c* | MSTRG.1368 | 35.0065 | 28.3161 | 42.5546 | 22.2387 | 27.6606 | 30.1679 | 0.3535 | 0.1940 |
| *cobO* | MSTRG. | 0.0601 | 0.2934 | 0.0294 | 1.8203 | 3.8446 | 0.1658 | 0.3770 | 0.1030 |
| *dxs1* | MSTRG. | 0.0079 | 0.1529 | 0.9392 | 2.2278 | 4.0266 | 0.0909 | 0.4040 | 0.1439 |
| *msrB* | MSTRG. | 4.6065 | 2.3526 | 1.6203 | 2.6092 | 8.4347 | 10.9944 | 0.4536 | 0.2135 |
| *aftC* | MSTRG. | 24.2094 | 15.7566 | 20.0102 | 16.3844 | 59.8269 | 48.5135 | 0.4578 | 0.1182 |
| *Rv3693* | MSTRG.2402 | 110.4871 | 98.7140 | 128.5447 | 96.1689 | 143.6528 | 94.4115 | 0.5084 | 0.3060 |
| *Rv1456c* | MSTRG.967 | 7.1502 | 0.3900 | 1.6742 | 0.9789 | 6.2539 | 5.7249 | 0.5310 | 0.4220 |
| *gnd2* | MSTRG.778 | 0.0227 | 0.0299 | 0.0220 | 0.0233 | 0.4470 | 2.6252 | 0.6040 | 0.3557 |
| *glgE* | MSTRG.921 | 0.0866 | 0.1232 | 0.1096 | 0.1296 | 0.2133 | 4.2428 | 0.6468 | 0.5208 |
| *mce2D* | MSTRG.427 | 4.6770 | 21.9995 | 5.8097 | 14.5262 | 27.8425 | 3.8571 | 0.6667 | 0.6580 |
| *echA15* | MSTRG.1812 | 1.2474 | 0.4642 | 0.8209 | 0.51340 | 1.9056 | 1.8844 | 0.7030 | 0.2421 |
| *galTa* | MSTRG.448 | 87.3898 | 67.4198 | 65.3668 | 74.8863 | 118.3477 | 93.8430 | 0.7188 | 0.0909 |
| *wag31* | MSTRG.1437 | 0.0159 | 0.01356 | 0.01236 | 0.0272 | 0.1847 | 1.6133 | 0.7264 | 0.4186 |

WT: wild-type; ∆*mtp*: *mtp*-gene knockout mutant; fold change (FC); Fragments per kilobase of transcript per million mapped reads FPKM). *p* ≤ 0.05 was considered significant.

| **Table S2.** Differentially expressed up- and down-regulated genes induced by the Δ*hbhA* deletion mutant relative to the wild-type. | | | | | | | | | |
| --- | --- | --- | --- | --- | --- | --- | --- | --- | --- |
| **Gene** | **Gene ID** | **FPKM**  **Δ*hbhA1*** | **FPKM Δ*hbhA2*** | **FPKM Δ*hbhA3*** | **FPKM WT1** | **FPKM WT2** | **FPKM WT3** | **FC** | ***p-value*** |
| **Up-regulated genes** | | | | | | | | | |
| *hspX* | MSTRG.1224 | 39.3371 | 1472.0340 | 1862.0740 | 101.5714 | 1119.7840 | 793.4222 | 6.0139 | 0.1005 |
| *Rv2030c* | MSTRG.1223 | 22.5371 | 463.7529 | 541.5175 | 90.9116 | 441.7668 | 216.1086 | 3.6106 | 0.0256 |
| *fdxA* | MSTRG.1207 | 64.5701 | 748.3990 | 1052.2810 | 184.5936 | 697.0797 | 461.0035 | 3.1517 | 0.0447 |
| *hrp1* | MSTRG.1891 | 27.5074 | 299.6852 | 515.6089 | 95.0092 | 370.4746 | 197.4117 | 2.9233 | 0.0317 |
| *Rv3134c* | MSTRG.2378 | 38.3891 | 247.9740 | 405.3703 | 110.1277 | 297.0371 | 190.0389 | 2.2525 | 0.0400 |
| *TB31.7* | MSTRG.1888 | 29.2302 | 262.4276 | 373.4244 | 122.8330 | 332.1042 | 162.2937 | 2.2477 | 0.0064 |
| *devS* | MSTRG.2377 | 54.8864 | 237.3781 | 324.0018 | 111.2764 | 257.3765 | 135.7297 | 2.2243 | 0.0021 |
| *devR* | MSTRG.2377 | 62.8539 | 276.2814 | 384.5554 | 128.8909 | 370.3603 | 167.6869 | 2.1301 | 0.0098 |
| *pfkB* | MSTRG.1222 | 22.1567 | 201.5600 | 209.1792 | 91.6447 | 223.4125 | 105.0169 | 2.0884 | 0.0044 |
| *PPE57* | MSTRG.2535 | 0.7628 | 2.0701 | 4.5503 | 1.0748 | 2.2135 | 1.2844 | 1.9092 | 0.0498 |
| *PE12* | MSTRG.618 | 0.0677 | 0.1238 | 0.6326 | 0.6452 | 0.0051 | 0.0327 | 1.8438 | 0.0413 |
| *Rv0482* | MSTRG.1421 | 11.2162 | 55.6094 | 93.1676 | 34.7253 | 77.1798 | 45.5266 | 1.8285 | 0.0357 |
| *secE2* | MSTRG.1336 | 24.2545 | 147.9241 | 146.6986 | 89.6788 | 149.4047 | 85.5972 | 1.7084 | 0.0286 |
| *Rv2028c* | MSTRG.1222 | 24.5516 | 176.5223 | 189.1088 | 106.3481 | 206.6830 | 123.2315 | 1.6359 | 0.0334 |
| *Rv3128c* | MSTRG.2373 | 58.4169 | 319.6930 | 426.4213 | 204.2645 | 399.3027 | 267.7664 | 1.6336 | 0.0455 |
| *serT* | MSTRG.2797 | 39.5135 | 209.1746 | 187.2323 | 125.1723 | 235.6404 | 118.3144 | 1.5994 | 0.0273 |
| *mpr17* | MSTRG.2596 | 88.5026 | 266.9240 | 211.1838 | 142.8192 | 234.9860 | 148.6242 | 1.5877 | 0.0391 |
| *Rv2005c* | MSTRG.1205 | 52.8105 | 215.9812 | 332.3335 | 146.4234 | 343.4680 | 193.5525 | 1.5858 | 0.0431 |
| *csoR* | MSTRG.484 | 83.5283 | 284.5811 | 254.0524 | 158.5629 | 276.4202 | 195.7747 | 1.5188 | 0.0344 |
| *Rv2004c* | MSTRG.1204 | 40.5525 | 152.9136 | 210.4625 | 105.3865 | 240.1092 | 128.9590 | 1.5112 | 0.0205 |
| *phoH2* | MSTRG.560 | 37.6987 | 3.3317 | 12.3612 | 3.5641 | 3.3945 | 0.3263 | 2.6035 | 0.3528 |
| *rpmG1* | MSTRG.1237 | 69.1233 | 441.5842 | 184.5275 | 110.2679 | 154.4569 | 159.5765 | 2.2300 | 0.1774 |
| *rpmB2* | MSTRG.1237 | 44.2660 | 290.4057 | 149.5706 | 91.7071 | 125.8018 | 111.1051 | 2.1400 | 0.1339 |
| *glnU* | MSTRG.2208 | 49.1144 | 495.3003 | 219.8441 | 193.0665 | 409.8250 | 166.4166 | 1.7589 | 0.2557 |
| *Rv2477c* | MSTRG.1850 | 13.4429 | 2.1132 | 3.6629 | 1.5439 | 0.0586 | 1.1175 | 1.7339 | 0.1003 |
| *eccA5* | MSTRG.1019 | 0.3350 | 1.8860 | 2.1258 | 0.5338 | 0.6457 | 1.2781 | 1.7330 | 0.1299 |
| *Rv1461* | MSTRG.841 | 20.7525 | 20.7107 | 20.3962 | 9.7493 | 19.9350 | 12.6493 | 1.6300 | 0.1429 |
| *glpQ1* | MSTRG.2863 | 406.8954 | 349.8250 | 428.1983 | 178.7321 | 442.3802 | 251.6407 | 1.6320 | 0.2558 |
| *rpmJ* | MSTRG.2522 | 6109.8420 | 2990.4840 | 2441.2010 | 1991.1210 | 1336.8920 | 1605.3010 | 1.4946 | 0.0227 |
| *PPE39* | MSTRG.1751 | 793.6209 | 727.9490 | 611.8809 | 449.4208 | 393.1288 | 451.1813 | 1.4904 | 0.0091 |
| *Rv2624c* | MSTRG.1889 | 19.6208 | 160.0454 | 213.4221 | 121.8138 | 254.5644 | 134.4763 | 1.4870 | 0.0520 |
| *Rv2885c* | MSTRG.2125 | 62.1975 | 233.5320 | 217.1311 | 139.4542 | 246.0377 | 167.1307 | 1.4727 | 0.0301 |
| *rsfB* | MSTRG.2699 | 96.6495 | 272.1294 | 240.7912 | 166.5467 | 293.4629 | 177.2220 | 1.4434 | 0.0328 |
| *infA* | MSTRG.2523 | 7975.3210 | 3616.8870 | 2862.0660 | 2234.5280 | 1638.3540 | 2183.8390 | 1.4366 | 0.0161 |
| *Rv2628* | MSTRG.1906 | 45.7256 | 171.2548 | 203.7106 | 125.7012 | 226.4752 | 137.7727 | 1.4206 | 0.0028 |
| *Rv3196A* | MSTRG.2292 | 62.6009 | 189.4726 | 188.2155 | 132.9760 | 211.9940 | 136.1313 | 1.3913 | 0.0059 |
| *leuX* | MSTRG.534 | 62.6009 | 189.4726 | 188.2155 | 132.9760 | 211.9940 | 136.1313 | 1.3481 | 0.0091 |
| *Rv2625c* | MSTRG.1890 | 32.5123 | 154.4425 | 181.5053 | 123.2851 | 246.4574 | 127.6836 | 1.3245 | 0.0011 |
| *atpB* | MSTRG.716 | 619.4113 | 280.9315 | 351.3754 | 277.6300 | 350.1990 | 246.6503 | 1.1059 | 0.7360 |
| *atpC* | MSTRG.723 | 181.5092 | 158.9081 | 182.5291 | 204.3908 | 203.4880 | 130.0632 | 1.0787 | 0.9901 |
| *ctaC* | MSTRG.1619 | 442.1451 | 402.8933 | 335.8707 | 271.1359 | 379.5308 | 288.6349 | 1.2327 | 0.3246 |
| **Down-regulated genes** | | | | | | | | | |
| *lpqV* | MSTRG.544 | 0.8223 | 0.0201 | 0.1388 | 0.0235 | 0.8290 | 0.0169 | 0.9360 | 0.8608 |
| *mtp* | MSTRG.2411 | 13.8123 | 56.1472 | 67.4912 | 118.7850 | 213.8307 | 112.2085 | 0.5334 | 0.0010 |
| *hbhA* | MSTRG.1416 | 2.4985 | 10.1726 | 5.9029 | 133.6487 | 251.4865 | 133.4202 | 0.0615 | 0.0015 |
| *cobO* | MSTRG.2135 | 0.0146 | 0.0253 | 0.0158 | 2.9687 | 4.6153 | 0.1896 | 0.3980 | 0.2375 |
| *groEL2* | MSTRG.1384 | 86.5363 | 72.2120 | 86.0036 | 156.5173 | 144.9753 | 86.5231 | 0.6515 | 0.2059 |
| *vapC38* | MSTRG.1831 | 135.5824 | 95.0031 | 106.8143 | 146.1758 | 186.3456 | 114.8229 | 0.7232 | 0.2565 |
| *Rv2650c* | MSTRG.1959 | 0.6453 | 1.4857 | 3.4969 | 4.1113 | 2.6736 | 4.2264 | 0.7248 | 0.4257 |
| *coaA* | MSTRG.562 | 1.0252 | 0.7830 | 1.7263 | 1.8262 | 3.5619 | 1.9224 | 0.7255 | 0.2712 |
| *vapB38* | MSTRG.18130 | 125.9363 | 89.61166 | 113.2298 | 123.6264 | 149.8421 | 145.1767 | 0.7371 | 0.1285 |
| *Rv2928* | MSTRG.2192 | 13.13711 | 6.9414 | 6.1818 | 10.8895 | 10.3194 | 13.2774 | 0.5706 | 0.0117 |
| *purA* | MSTRG.1319 | 2.0739 | 5.1115 | 4.7807 | 8.3250 | 8.8787 | 8.3839 | 0.6541 | 0.0302 |
| *Rv2642* | MSTRG.1955 | 22.9796 | 77.0873 | 63.4679 | 108.3547 | 180.6468 | 104.6858 | 0.6439 | 0.0378 |
| *Rv1353c* | MSTRG.768 | 0.0040 | 0.0083 | 0.0436 | 0.8934 | 1.1818 | 0.4659 | 0.5893 | 0.0392 |
| *Rv1082* | MSTRG.560 | 0.9778 | 0.0125 | 0.3588 | 1.8043 | 1.2380 | 0.9295 | 0.4937 | 0.0441 |
| *atpE* | MSTRG.717 | 497.4536 | 272.6431 | 292.0510 | 288.2544 | 315.1299 | 261.9014 | 0.9823 | 0.8800 |
| *atpF* | MSTRG.718 | 576.7258 | 239.1770 | 317.6221 | 341.2621 | 317.7958 | 267.4504 | 0.8909 | 0.5828 |
| *atpH* | MSTRG.719 | 479.0136 | 183.4726 | 286.7517 | 296.8543 | 276.9630 | 178.0221 | 0.9533 | 0.8562 |
| *atpA* | MSTRG.720 | 272.3081 | 151.0631 | 198.8215 | 215.5649 | 217.8918 | 177.5110 | 0.8831 | 0.4656 |
| *atpG* | MSTRG.721 | 338.7036 | 156.7466 | 184.8222 | 216.9375 | 201.4659 | 135.6632 | 0.9275 | 0.8030 |
| *atpD* | MSTRG.722 | 233.7669 | 130.5056 | 161.8864 | 190.5478 | 187.0747 | 138.3515 | 0.8535 | 0.5129 |

WT: wild-type; ∆*hbhA*: *hbhA*-gene knockout mutant; fold change (FC); Fragments per kilobase of transcript per million mapped reads (FPKM). *p* ≤ 0.05.

| **Table S3.** Differentially expressed up- and down-regulated genes induced by the *Δmtp-hbhA* deletion mutant relative to the wild-type. | | | | | | | | | |
| --- | --- | --- | --- | --- | --- | --- | --- | --- | --- |
| **Gene** | **Gene ID** | **FPKM *Δmtp-hbhA1*** | **FPKM Δ *Δmtp-hbhA 2*** | **FPKM Δ *Δmtp-hbhA 3*** | **FPKM WT1** | **FPKM WT2** | **FPKM WT3** | **FC** | ***p-value*** |
| **Up-regulated genes** | | | | | | | | | |
| *ASdes* | MSTRG.592 | 8.4995 | 10.6641 | 8.0976 | 6.9160 | 4.4360 | 5.7415 | 1.7061 | 0.0153 |
| *PPE59* | MSTRG.2473 | 1.6320 | 1.8753 | 1.1342 | 0.4880 | 0.4980 | 0.4239 | 1.6938 | 0.0207 |
| *moaA1* | MSTRG.2244 | 1.0902 | 1.2581 | 1.2982 | 0.5428 | 0.4037 | 0.8175 | 1.5022 | 0.0099 |
| *lpdA* | MSTRG.2397 | 188.7717 | 182.7458 | 189.6203 | 83.5332 | 150.4910 | 82.7390 | 1.4947 | 0.0223 |
| *atpB* | MSTRG.971 | 268.6685 | 433.0232 | 264.2791 | 276.4777 | 348.9817 | 244.4468 | 1.23843 | 0.9287 |
| *atpH* | MSTRG.974 | 269.8682 | 318.5572 | 26.8346 | 295.6222 | 276.0003 | 176.4317 | 1.0317 | 0.9088 |
| *atpC* | MSTRG.978 | 195.2074 | 215.6235 | 172.4360 | 203.5425 | 202.7807 | 128.9012 | 1.0363 | 0.8708 |
| *secE2* | MSTRG.283 | 240.2260 | 145.5616 | 150.7794 | 89.3066 | 148.8853 | 84.8324 | 1.2976 | 0.1164 |
| *Rv0986* | MSTRG.731 | 2.4821 | 7.0710 | 2.9423 | 2.5856 | 2.2333 | 3.0763 | 1.5061 | 0.2980 |
| *Rv0987* | MSTRG.731 | 20.7947 | 61.9206 | 30.0647 | 19.2461 | 20.4594 | 24.7519 | 1.7552 | 0.2548 |
| *iniB* | MSTRG.240 | 4.7448 | 2.1040 | 0.0235 | 0.0063 | 0.0099 | 2.6695 | 1.7669 | 0.5627 |
| *mas* | MSTRG.2128 | 2.0120 | 1.9255 | 0.0510 | 0.0151 | 0.2265 | 0.0126 | 1.7009 | 0.3126 |
| *efpA* | MSTRG.2058 | 0.0558 | 6.9563 | 8.9505 | 0.05138 | 4.9211 | 0.2137 | 1.6689 | 0.6852 |
| *metE* | MSTRG.840 | 7.5315 | 4.6598 | 1.2129 | 5.1588 | 1.2727 | 1.4474 | 1.6042 | 0.5309 |
| *cysG* | MSTRG.2058 | 0.8127 | 1.4511 | 0.0179 | 0.0190 | 0.0260 | 0.0152 | 1.5951 | 0.2642 |
| *Rv2652c* | MSTRG.1918 | 2.6218 | 1.2246 | 2.3220 | 1.1287 | 0.8749 | 1.1390 | 1.4688 | 0.1603 |
| *Rv3256c* | MSTRG.2360 | 191.0150 | 130.4797 | 183.4209 | 85.5605 | 118.3392 | 103.8679 | 1.4514 | 0.0895 |
| **Down-regulated genes** | | | | | | | | | |
| *lpqV* | MSTRG.798 | 0.9308 | 0.0152 | 0.0624 | 0.0234 | 0.8262 | 0.0168 | 0.7737 | 0.2140 |
| *hbhA* | MSTRG.363 | 6.2477 | 7.0330 | 8.8305 | 133.0940 | 250.6123 | 132.2282 | 0.0415 | 0.0005 |
| *Rv3312A/mtp* | MSTRG.2406 | 79.2700 | 70.9598 | 95.7475 | 118.2919 | 213.0874 | 111.2061 | 0.4706 | 0.0192 |
| *Rv3326* | MSTRG.2414 | 18.2786 | 14.8242 | 13.7698 | 13.6232 | 31.5973 | 19.0127 | 0.5989 | 0.0157 |
| *Rv2815c* | MSTRG.2056 | 45.0767 | 39.2355 | 36.8853 | 34.0839 | 86.2919 | 40.3159 | 0.6002 | 0.0080 |
| *Rv3475* | MSTRG.2503 | 24.8611 | 18.7321 | 18.6580 | 17.8368 | 37.4701 | 26.2625 | 0.6341 | 0.0356 |
| *Rv2042c* | MSTRG.1473 | 0.0287 | 0.0030 | 0.0973 | 0.03611 | 0.9366 | 0.1866 | 0.6424 | 0.0267 |
| *mce2D* | MSTRG.453 | 5.4070 | 4.3226 | 22.2101 | 14.5661 | 27.9559 | 3.8679 | 0.5149 | 0.4880 |
| *PE_PGRS26* | MSTRG.1046 | 0.4301 | 0.0786 | 0.2923 | 0.0437 | 1.17542 | 1.7504 | 0.5960 | 0.2540 |
| *Rv1377c* | MSTRG.1022 | 1.4386 | 0.5913 | 1.019 | 1.6965 | 2.7604 | 0.9288 | 0.5979 | 0.0610 |
| *aprA* | MSTRG.1736 | 3.2462 | 10.9635 | 0.4670 | 10.4472 | 7.8829 | 0.9561 | 0.6108 | 0.6755 |
| *Rv0487* | MSTRG.368 | 0.2206 | 3.9921 | 0.5362 | 1.0385 | 4.92002 | 0.1583 | 0.6253 | 0.5806 |
| *Rv3475* | MSTRG.2503 | 24.8611 | 18.7321 | 18.6580 | 17.8368 | 37.4701 | 26.2625 | 0.6341 | 0.0356 |
| *ncrMT1234* | MSTRG.888 | 2669.317 | 1835.4830 | 561.0939 | 3278.2630 | 1789.6270 | 1812.0800 | 0.6352 | 0.5499 |
| *Rv1096* | MSTRG.810 | 0.0144 | 1.2404 | 0.1046 | 1.7968 | 1.2337 | 0.9212 | 0.6470 | 0.2996 |
| *atpE* | MSTRG.972 | 304.0207 | 370.4341 | 219.3473 | 287.0580 | 314.0345 | 259.5616 | 0.9670 | 0.8807 |
| *atpF* | MSTRG.973 | 298.2995 | 369.4603 | 241.3163 | 339.8457 | 316.6912 | 265.0610 | 0.9673 | 0.8717 |
| *atpA* | MSTRG.975 | 196.2348 | 224.0662 | 162.2335 | 214.6702 | 217.1344 | 175.9252 | 0.9614 | 0.6166 |
| *atpG* | MSTRG.976 | 184.6530 | 218.6242 | 152.6178 | 216.0371 | 200.7656 | 134.4512 | 0.9738 | 0.9161 |
| *atpD* | MSTRG.977 | 159.4179 | 187.3604 | 161.2693 | 189.7569 | 186.4244 | 137.1155 | 0.9885 | 0.8455 |

WT: wild-type; ∆*mtp-hbhA*: *mtp-hbhA*-gene knockout mutant; fold change (FC); Fragments per kilobase of transcript per million mapped reads (FPKM). *p* ≤ 0.05 was considered significant.

## RT-qPCR

**Table S4.** The RT-qPCR expression means and standard deviations for each gene for the *M. tuberculosis* WT. deletion mutants. and their respective complements

WT: wild-type; ∆*mtp*: *mtp*-gene knockout mutant; ∆*hbhA*: *hbhA*-gene knockout mutant; ∆*mtp-hbhA*: *mtp-hbhA*-gene knockout mutant. SD: standard deviation.

|  | **Wild-type** | | **∆*mtp*** | | ***mtp-*complement** | | **∆*hbhA*** | | ***hbhA-*complement** | | **∆*mtp-hbhA*** | | ***mtp-hbhA***  **complement** | | |
| --- | --- | --- | --- | --- | --- | --- | --- | --- | --- | --- | --- | --- | --- | --- | --- |
| **Gene** | Mean | SD | Mean | SD | Mean | SD | Mean | SD | Mean | SD | Mean | SD | Mean | SD |  |
| ***atpB*** | 0.6680 | 0.0120 | 0.7640 | 0.0090 | 0.6850 | 0.0150 | 0.7800 | 0.0200 | 0.6990 | 0.0100 | 0.7990 | 0.0510 | 0.7140 | 0.0120 |  |
| ***atpD*** | 0.6210 | 0.0240 | 0.7050 | 0.0180 | 0.6730 | 0.0170 | 0.6900 | 0.0100 | 0.6700 | 0.0270 | 0.7300 | 0.0460 | 0.6930 | 0.0540 |  |
| ***atpE*** | 0.6790 | 0.0130 | 0.7390 | 0.0330 | 0.7040 | 0.0110 | 0.7300 | 0.0300 | 0.6860 | 0.0110 | 0.7770 | 0.0410 | 0.6890 | 0.0130 |  |
| ***atpF*** | 0.6690 | 0.0060 | 0.7510 | 0.0090 | 0.6930 | 0.0290 | 0.7500 | 0.0200 | 0.6720 | 0.0180 | 0.8000 | 0.0440 | 0.6970 | 0.0080 |  |
| ***atpH*** | 0.6440 | 0.0290 | 0.7430 | 0.0190 | 0.6970 | 0.0210 | 0.7700 | 0.0400 | 0.6890 | 0.0060 | 0.7880 | 0.0530 | 0.7600 | 0.0440 |  |
| ***lpqV*** | 0.5800 | 0.0710 | 0.6350 | 0.0160 | 0.6100 | 0.0100 | 0.6400 | 0.0100 | 0.6090 | 0.0100 | 0.7140 | 0.0790 | 0.6020 | 0.0080 |  |
| ***secE2*** | 0.6270 | 0.0060 | 0.6690 | 0.0030 | 0.6480 | 0.0060 | 0.7500 | 0.0500 | 0.6410 | 0.0030 | 0.7590 | 0.0630 | 0.6280 | 0.0070 |  |
| ***Rv0986*** | 0.5870 | 0.0080 | 0.6270 | 0.0150 | 0.5960 | 0.0400 | 0.6200 | 0.0100 | 0.5870 | 0.0020 | 0.7840 | 0.0480 | 0.6000 | 0.0070 |  |
| ***Rv0987*** | 0.5610 | 0.0210 | 0.6110 | 0.0060 | 0.5690 | 0.0170 | 0.6100 | 0.0100 | 0.5640 | 0.0070 | 0.7650 | 0.0720 | 0.5860 | 0.0140 |  |
